# Sequence-Specific Recognition of DNA by Proteins: Binding Motifs Discovered Using a Novel Statistical/Computational Analysis

**DOI:** 10.1371/journal.pone.0158704

**Published:** 2016-07-06

**Authors:** David Jakubec, Roman A. Laskowski, Jiri Vondrasek

**Affiliations:** 1 Institute of Organic Chemistry and Biochemistry, Prague 6, Czech Republic; 2 Department of Physical and Macromolecular Chemistry, Faculty of Science, Charles University in Prague, Prague 2, Czech Republic; 3 EMBL-EBI, Wellcome Trust Genome Campus, Hinxton, Cambridge, United Kingdom; Indian Institute of Science, INDIA

## Abstract

Decades of intensive experimental studies of the recognition of DNA sequences by proteins have provided us with a view of a diverse and complicated world in which few to no features are shared between individual DNA-binding protein families. The originally conceived direct readout of DNA residue sequences by amino acid side chains offers very limited capacity for sequence recognition, while the effects of the dynamic properties of the interacting partners remain difficult to quantify and almost impossible to generalise. In this work we investigated the energetic characteristics of all DNA residue—amino acid side chain combinations in the conformations found at the interaction interface in a very large set of protein—DNA complexes by the means of empirical potential-based calculations. General specificity-defining criteria were derived and utilised to look beyond the binding motifs considered in previous studies. Linking energetic favourability to the observed geometrical preferences, our approach reveals several additional amino acid motifs which can distinguish between individual DNA bases. Our results remained valid in environments with various dielectric properties.

## Introduction

Interactions of deoxyribonucleic acid (DNA) with proteins form the basis of several essential processes of cellular physiology. These interactions display various levels of specificity towards the designated DNA base sequences. For example, the interactions of DNA with repair enzymes must display low sequence preferences if genome integrity is to be maintained [1–3], and histone proteins involved in nucleosomes have been shown to promote non-specific association with nucleic acids [4–6]. On the other hand, for processess such as regulation of gene expression by transcription factor proteins, DNA sequence recognition with high specificity is critical.

Experimental studies utilising X-ray diffraction or nuclear magnetic resonance spectroscopy have been actively used to explore atomic-level details of proteins, nucleic acids, and their complexes for more than half a century. The RSCB Protein Data Bank (PDB) currently hold over 3,000 structures of protein—DNA complexes obtained from a variety of organisms by a range of experimental methods [7, 8]. Structural and biochemical studies of proteins and their cognate DNA sequences were recently performed for some whole organisms [9, 10].

Years of analyses of experimental structures have revealed two principal contributions to the process of specific DNA sequence recognition. The base readout mechanism involves local interactions between the protein DNA-binding domain and the target base sequence, predominantly in the form of a matching pattern of bidentate hydrogen bond donor and acceptor groups [11]. Asparagine and glutamine are capable of distinguishing between the Hoogsteen edge of adenine and the other DNA bases, while specific recognition of guanine by these amino acids is possible from the sugar edge. In addition, arginine can recognise the Hoogsteen edge of guanine [12].

However, the local, linear model of DNA sequence recognition by a complementary pattern of hydrogen bond donor and acceptor groups was found to be incomplete. In some protein—DNA complexes, the readout of the nucleic acid shape can be equally, or even more, important [11]. The DNA often adapts non-canonical forms in the interaction region, and its propensity to form various local distortions is dependent on a larger base sequence context [13–18]. GC-rich regions of the genome have a predisposition to adopt A-like conformations, while high AT content results in a narrowing of the minor groove, creating more negative electrostatic potential, a feature universally recognised by arginine side chains [17, 19].

Base readout and DNA shape recognition are both utilised to some extent in the majority of protein—DNA complexes. The formation of hydrogen bonds between the protein and the nucleic acid can induce a sequence-dependent distortion, which may, in turn, enable the formation of a new set of specific contacts. Therefore, the two modes cannot be separated if a complete description of the recognition process is to be obtained [13, 16, 20]. Structural data suggest that while the amino acid composition of the interface often provides sufficient information to distinguish between individual families of transcription factors, subtle differences in the dynamic properties of the cognate DNA region can guide the higher-resolution recognition by specific members of a single protein family [11, 16, 21].

In spite of these insights, no recognition code applicable to all protein families has been described to date, although recognition codes for a few genome editing enzymes are known [11, 22]. Current knowledge of the non-local properties of large blocks of DNA residues is lacking, while the dynamic aspects of the protein—nucleic acid interaction remain difficult to investigate both theoretically and experimentally. On the other hand, studies of amino acid—DNA base interactions, which probe atomic-level details of the direct readout mechanism, can readily be performed. While limited experimental data on these interactions are available [23, 24], computer technology has enabled analyses that simultaneously investigate the binding mechanism in thousands of protein—DNA complexes. Indeed, a substantial part of our understanding of the interactions involving these elementary biomolecular building blocks has been derived from studies utilising bioinformatics and other computational approaches.

The pioneering work of Berg and von Hippel combining the experimental results available at that time with a statistical mechanical framework offered one of the first rigorous theoretical treatments of specificity in protein—DNA interactions [25].

Mandel-Gutfreund and Margalit were among the first to utilise a data set of three-dimensional structures to derive contact potentials for prediction of protein—DNA interaction sites. They found that amino acids that carry bidentate hydrogen bond donor and acceptor groups and therefore enable DNA base recognition in a one-to-one manner, are strongly favoured at the interface [26]. Luscombe *et al.* also observed significant correlations between the populations of the same amino acid side chains and their cognate DNA bases. In addition, some other contacts that did not feature bidentate hydrogen bonding motifs were dubbed “context-specific.” Although the amino acids involved could not by themselves distinguish between individual DNA bases, their presence at the interface was deemed essential for the stabilisation of the respective complexes [27].

Dror *et al.* have recently performed a detailed analysis of the binding mechanisms *via* which homeodomains recognise their DNA binding sites. By combining protein and DNA sequence and shape covariation analysis with binding data obtained from high-throughput methods, specific positions containing amino acids facilitating DNA shape recognition were uncovered in the N-terminal tail of the homeodomain [28]. This study thus highlighted the importance of DNA geometry in binding site recognition. Further effort was also made in the study of DNA shape readout by other transcription factor protein families [29].

Many online databases focusing on protein—DNA complexes have been established over the past decade, their functionality ranging from simple repositories to sites offering complex analytical tools [27, 30–33].

The aforementioned studies of base readout have been based on statistical analysis and decomposition of the interfacial region of existing experimental structures. This treatment does not explicitly consider the physico-chemical characteristics of the interacting molecules. A different approach, utilising the methods of computational chemistry, is possible. Indeed, theoretical studies to calculate the interaction properties of amino acid—DNA base dimers have been conducted at both the quantum mechanical (QM) and empirical potential levels [34–40].

In this work, we combine an approach based on statistical analysis of existing experimental structures of protein—DNA complexes with molecular mechanical (MM) calculations. We have recently shown the very satisfactory performance of these computational methods when calculating the interaction energies of dimers of amino acids with DNA bases *in vacuo* in comparison with mid-level DFT calculations [41], and a similar level of agreement has been observed in comparison with high-level correlated QM results [42]. Here, we probe the explicit contribution of the charged phosphate group to the recognition of DNA bases on a physical basis for the first time. We weighted the importance of various interaction motifs based on their relative abundance in the structures of real protein—DNA complexes. This allowed us to view the interaction specificity as a function of the energetic favourability and geometrical conservation of the binding motifs. Finally, we tested the validity of the observed interaction preferences in different dielectric environments in an effort to effectively capture the intricate electric properties of the protein—nucleic acid interface.

## Materials and Methods

### Data set preparation

A set of 1,584 structures of protein—DNA complexes solved by X-ray crystallography to a resolution better than 2.5 Å and with an R-factor no worse than 0.25 was obtained from the PDB in April 2014 and refined using the PISCES sequence culling server [7, 43]. Only the entries containing at least one double stranded DNA region consisting of at least 4 base pairs were considered. When multiple identical polypeptide chains were included in the PDB structures, such as in the complexes of homomultimeric transcription factors, only a single (alphabetically first) protein chain in complex with DNA was retained. The structures of hetero-*N*-meric protein complexes were separated into *N* independent entries, each one containing a single protein chain interacting with a replica of the recognised DNA double helix. These proteins were further analysed independently, *i.e.*, during the sequence homology assessment (see below). In total, 1,737 unique polypeptide chains in complex with their cognate DNA sequences comprised the data set.

From each of these complexes, all interacting amino acid—2’-deoxyribonucleoside 5’-monophosphate (dNMP) dimers were extracted as follows. The interaction between the residues was defined based on the distance-based criteria utilised in the construction of the Atlas of Protein Side Chain Interactions [44]: when the distance between any amino acid reference atom and any DNA residue heavy atom was less than 1.0 Å plus the sum of the atoms’ van der Waals radii, a contact was defined [45]. Three reference atoms were selected for each amino acid and coincide with the residue’s characteristic side chain group atoms [45]. Pairs in which the nucleotide would originate from the 5’ end of the DNA strand were discarded, as these residues naturally lack the 5’ phosphate group. No other distinctions were made between the different nucleotide conformers. A total of 47,480 amino acid—dNMP dimers were obtained this way.

**Fig 1:**
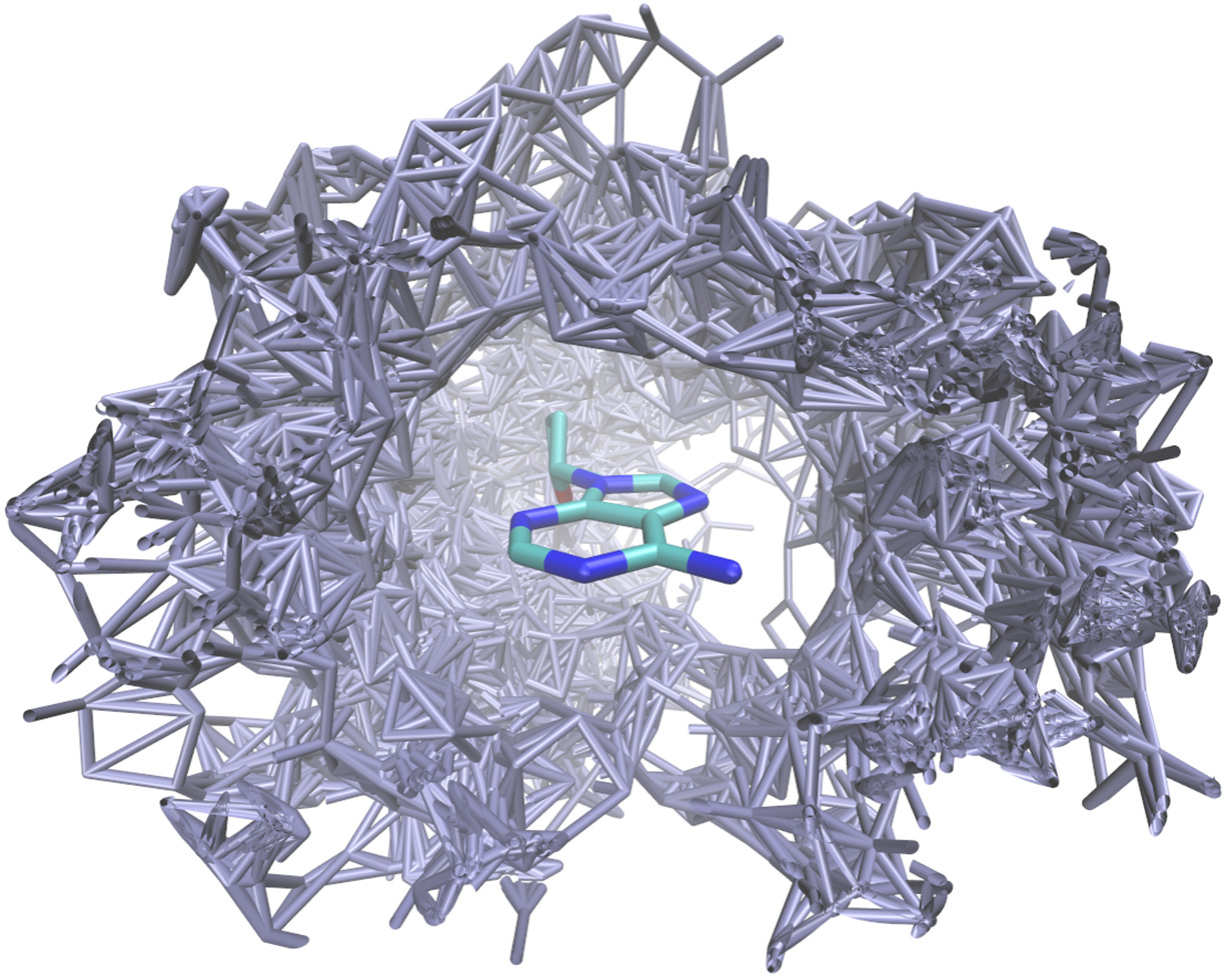
Distribution of asparagine side chains (gray) around an adenosine nucleoside. All molecular graphics were created using using VMD-1.9.2 [46].

When one geometrically transforms all dimers containing a certain amino acid—dNMP combination (for example, all deoxyadenosine 5’-monophosphate [dAMP]—asparagine contacts) into an appropriately chosen common frame of reference, a three-dimensional distribution of the amino acid residues around the nucleotide is obtained (Fig 1). These transformations were performed by minimising the root mean square deviation (RMSD) of the DNA base heavy atoms between all dimers of a particular type.

#### Detection of the clusters

**Fig 2:**
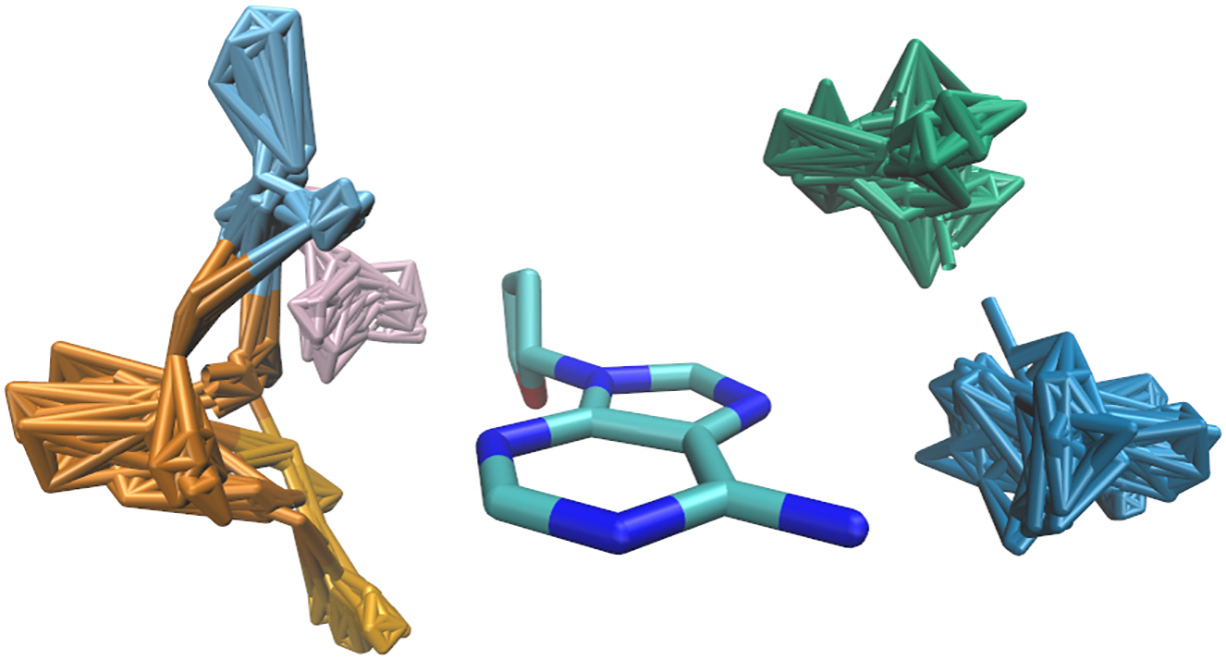
Side chain clusters identified in the distribution shown in Fig 1. The clusters are shown in colours (dark blue, vermillion, light blue, pink, green, orange).

As illustrated in Fig 2, the directional nature of some non-covalent interaction modes, notably hydrogen bonds, leads to the clustering of amino acid residues relative to the nucleotide in 3D [27, 41]. The rigorous identification of these clusters has previously been described in detail [41]. In brief, after all amino acid—dNMP dimers of a certain type had been transformed to superpose the DNA bases as described above, we picked out each amino acid in turn and calculated the RMSD between its reference atoms and the reference atoms of all other amino acids in the respective distribution. The amino acid for which the number of contacts with RMSD less than 1.5 Å was the largest was then recognised as a cluster representative and, together with its RMSD-derived neighbours (the cluster), taken out of the distribution. This process was repeated until 6 clusters had been identified in each distribution, or until the last cluster isolated was too sparsely populated to be considered significant. A total of 12,935 dimers were found within some of the 469 clusters [41]. It should be noted that the absolute number of contacts found in the clusters varied greatly between the various amino acid—DNA residue dimer types. While only few tens of contacts formed the clusters in the majority of distributions involving non-polar amino acids, up to several hundreds of contacts comprised the clusters in dimers which are known to form motifs significant for the process of direct sequence recognition (*i.e.*, arginine—guanine).

#### Treating the data set bias

While the redundant polypeptide chains corresponding to identical protein units within individual PDB files had already been discarded (see the treatment of homomultimeric proteins above), we had not at this point investigated the sequence identity of entries originating from different PDB structures. The high sequence similarity across different PDB entries would introduce bias into the data set, as the contacts originating from homologous protein structures would appear overpopulated compared to the contacts extracted from protein families for which few structures are currently available.

The bias was treated as follows. First, a global sequence alignment utilising the Needleman-Wunsch algorithm [47] and using the BLOSUM62 substitution matrix [48] was performed for all unique pairs of protein chains. The package *needle*, available in the EMBOSS-6.4.0.0 molecular biology suite, was used to perform these alignments [49]. Having obtained the sequence identity score for all pairs of protein chains, we constructed a set in which the sequence identity of any two proteins was less than 100%, *i.e.*, we removed the entries containing identical proteins.

Furthermore, we generated three additional sets of protein—DNA complexes, ones in which the sequence identity of any pair of protein chains was less than 30%, 90%, or 95%. These sets were generated as follows. For each of the 1,737 polypeptide chains, a list of proteins having sequence identity greater or equal to *X*% (*X* = 30, 90, 95) was compiled. These lists were then merged for each *X* to create a total list of homologous structures at the particular sequence identity level. The complements of these total lists are the sets of structures for which the sequence identity of any pair of protein chains is less than *X*%. The sets should be viewed as a more-or-less random selection of protein—DNA complexes satisfying the maximum sequence identity criteria; the randomness is limited by the fact that from each subset of homologous proteins, the alphanumerical order of the PDB identificators determined our selection. In fact, the same “randomness” was used in the construction of the set containing non-identical protein chains; however, in this case, the only difference between the PDB entries is the unlikely variation in the DNA sequences.

Table 1 presents the numbers of amino acid—dNMP dimers found for individual DNA base types. The populations of the sets constructed at the various maximum sequence identity levels are shown. The removal of identical protein entries discards over half of the available dimers (only 21,709 dimers remain from the original 47,480 dimers observed before the sequence identity culling was applied), while relatively little difference is seen when comparing the numbers of contacts found at the 30% and 90% maximum sequence identity levels. This suggests that the bias in the original set was caused by several overpopulated protein families, and that the these redundant entries were already discarded at the 90% sequence identity level. The difference between the populations obtained at 90% and 95% identity levels is negligible and thus not shown. Moreover, the relative populations of the clusters were struck harder by the treatment of bias. For example, from the 12,935 dimers that comprised the clusters before the sequence identity culling, only 3,897 remain after discarding structures containing 100% identical proteins. This can be rationalised by the fact that the discarded homologous structures were more likely to provide geometrically similar contacts. The percentages of contacts found in the clusters at each sequence identity level are shown in parentheses in Table 1.

**Table 1:** Number of contacts involving individual DNA base types after redundant entries had been discarded.

Seq. identity^†^	30%	90%	100%
**Adenine**	2,137 (7.9%)	3,087 (11.5%)	5,200 (18.5%)
**Guanine**	2,477 (6.9%)	3,411 (8.2%)	6,237 (16.7%)
**Cytosine**	2,007 (8.0%)	2,783 (10.0%)	4,899 (19.4%)
**Thymine**	2,305 (9.0%)	3,224 (11.5%)	5,373 (17.5%)
**Total**	8,926 (7.9%)	12,505 (10.3%)	21,709 (18.0%)

The numbers in parentheses indicate the proportion of contacts found in the clusters.

^†^—indicates that the mutual identity of any pair of sequences in the set is less than *X*%.

#### Identification of the contacts with DNA bases

The previously described procedure led to the retrieval of dimers in which the amino acid may be found in proximity to any of the dNMP’s base, 2’-deoxyribose, or phosphate moieties. To quantitatively assess the contribution of the DNA backbone groups to the interaction specificity, we isolated a subset of contacts in which the amino acids interact directly with the DNA base moiety. These contacts were again identified based on distance-dependent criteria: if the distance between any amino acid and any DNA base atom was found less than 1.0 Å plus the sum of the atoms’ van der Waals radii, the dimer was labelled as containing a base-directed interaction. Table 2 presents for various maximum sequence identity levels the number of base-directed contacts found in the distributions containing the respective DNA base types.

**Table 2:** As Table 1, but only including contacts in which the amino acid interacts directly with the DNA base.

Seq. identity^†^	30%	90%	100%
**Adenine**	1,080 (11.6%)	1,548 (17.1%)	2,462 (22.2%)
**Guanine**	1,313 (10.2%)	1,761 (11.5%)	3,011 (17.2%)
**Cytosine**	1,000 (9.5%)	1,358 (11.6%)	2,213 (18.9%)
**Thymine**	1,359 (10.7%)	1,879 (13.6%)	2,886 (17.2%)
**Total**	4,752 (10.5%)	6,546 (13.4%)	10,572 (18.7%)

The numbers in parentheses indicate the proportion of contacts found in the clusters.

^†^—indicates that the mutual identity of any pair of sequences in the set is less than *X*%.

We thus obtained two sets of contacts: the first contains all amino acid—dNMP dimers found in the respective non-redundant sets of protein—DNA complexes, while the second constitutes a subset of the former, containing only those dimers in which the amino acid interacts directly with the base moiety. In the framework of the pair-wise additive empirical calculations (see below), it was possible to investigate the exact interaction energy contribution of the interaction with the base moiety to the total interaction energy. To this end, we created two additional sets of contacts by duplicating the former and stripping their sugar-phosphate moieties. The energy of interaction between the amino acid and the sequence-specifying base moiety can be obtained from these dimers. Moreover, direct comparison with the contacts involving dNMPs can be made, revealing the quantitative contribution of the DNA backbone to the recognition process.

#### System preparation

The procedure atomising the interactions between proteins and DNA into the pairs of interacting residues described above led to the retrieval of amino acid—dNMP dimers. For multiple reasons, we decided to get rid of the atoms constituing the protein backbone groups. The inclusion of the C_*α*_ amide and carbonyl groups would introduce charged species into the molecule, greatly complicating the interpretation of the gas phase interaction energies. Second, each peptide bond group would have to be capped, creating intra- and intermolecular interactions that do not exist in nature. Finally, the properties of the atoms constituing the protein backbone are the same in each standard *α*-amino acid. Therefore, the binding motifs involving the peptide bond groups can hardly be viewed as representative of some preferred interaction mode between a specific amino acid—DNA residue dimers.

Therefore, we replaced the peptide bond carbonyl and amide groups of the amino acid with hydrogen atoms in each amino acid—DNA residue dimer. This process is consistent with those described in other works on the interactions of amino acids [39, 40, 50]. The result of this geometry culling is called the C_*α*_ representation of the amino acid, in which a methyl group caps the C_*β*_ atom. In the case of proline, only the carboxyl group was removed and the five-membered heterocycle remained [41].

Due to the way nucleic acid residues are labelled in PDB structures, the extraction of the *N*th DNA nucleotide resulted in the phosphate moieties lacking the O3’ oxygen atom belonging to 2’-deoxyribose of the immediately preceeding (*N* − 1)th residue. This oxygen atom was added at the end of a vector of length 1.610 Å originating at the P atom and perpendicular to the plane containing the atoms OP1, OP2, and O5’. The specific length was chosen because it represents the equilibrium bond length between the two atoms in the Amber94 force field [51].

As the dimers were extracted from X-ray structures only, no hydrogen atoms were originally present. This problem was remedied utilising a custom UCSF Chimera-1.8.1 [52] script, to add the hydrogen atoms to both the amino acid and DNA residues for all contacts. Given the C_*α*_ representations, proline was modelled as a neutral tetrahydropyrrole and glycine as methane. Despite the software being able to decide on the correct protonation based on the local environment [52], histidine was protonated on N_*ε*_ in all contacts, as the population of N_*δ*_-protonated side chains was insufficient for their separate analysis. Guanine and cytosine were represented by their dominant keto forms, and adenine and thymine by the dominant amino forms. Guanine was set to be protonated on the N1 atom instead of N3. In the two sets of contacts with isolated DNA bases, hydrogen atoms were added to N9 or N1 atoms in purine or pyrimidine bases, respectively. A single hydrogen was added to the O3’ atom of the phosphate group.

### Interaction energy determination

As noted in the Introduction, the total number of amino acid—DNA residue dimers in all four sets of contacts (over 60,000) and the size of some complexes (over 60 atoms) heavily favour the use of MM techniques over QM calculations. We have already shown the very satisfactory performance of MM methods for the calculation of interaction energies of amino acid side chain—DNA base dimers [41]. Therefore, we followed the same computational protocol to calculate interaction energies of the extended complexes presented in this work.

#### Derivation of the missing parameters

The parameters used for the atoms of the C_*α*_ representations of amino acids, the atoms of the isolated nitrogenous bases, and atoms of the dNMPs were those derived for the Amber94 or, where applicable, Amber99SB-ILDN force field [51, 53]. The atoms not present in the force field (C_*α*_ hydrogen atoms, proline H1 atom, H1 and H9 atoms in isolated pyrimidine and purine bases, respectively, and the phosphate group’s O3’ and its attached hydrogen atom) had their non-electrostatic parameters assigned from chemically equivalent atom types. The constants of interactions between bonded atoms not present in the original force field were manually added based on the functional similarities of the atoms involved.

Partial atomic charges were assigned to the added hydrogen atoms manually in order to retain an integral total charge of each residue: +1.0 for arginine and lysine; −1.0 for aspartate, glutamate, and dNMPs; and 0.0 for the remaining amino acids and isolated DNA bases. Only the dominant forms of the species at pH = 7 were considered. When multiple hydrogen atoms were added, the charges were split symmetrically.

#### Computational protocol

The interaction energy calculations were performed using the supermolecular approach. First, the amino acid—dNMP (base) dimer had its hydrogen atoms’ positions optimised using conjugate gradient energy minimisation while the heavy (non-hydrogen) atoms were confined to their original positions. Single point energy was then calculated on this optimised dimer geometry. The dimer was then split and a single point energy calculation was immediately calculated for monomer. Hydrogen atom positions were optimised for each monomer by itself, and then a single point energy was calculated. The difference between the single point energy of the monomer after it had been isolated from the complex and after it was optimised by itself is the deformation energy of that monomer. The interaction energy was then calculated as the difference between the single point energy of the optimised complex and the sum of the single point energies of the monomers present in the non-optimised, dimer conformation, plus the sum of the monomers’ deformation energies. All MM interaction energy calculations and geometry optimisations were performed using GROMACS-4.5.5 compiled in double precision [54].

#### Solvation effects

To introduce biological relevance to the interaction energy calculations, we included the effects of the surrounding water environment. Molecular dynamics (MD) simulations are usually performed using discrete water models in which each water molecule is treated explicitly. This representation of the solvent is not suitable for the interaction energy calculations presented here. In particular, the requirement of the constrained geometry of the solute would introduce artificial energy gaps between the dimer and monomer conformations of the interacting molecules when solvent relaxation was taken into account.

An alternative approach is to part with the discrete representation of the water molecules and to treat the solvent as a continuous dielectric environment. The electrostatic interaction of the solute with this implicit solvent model is described by the Poisson equation. Given the technical difficulties of solving this differential equation, various approximations to the (linearised) Poisson equation have been derived. Among these, the generalised Born (GB) formalism is the most widely used in simulations of biomolecules [55–57]. Combined with a term accounting for the disruption of the solvent structure due to the presence of the molecule proportional to its surface area (SA), the GB approach can be used to calculate the free energy of solvation of any molecule for which the set of atomic Born radii are known [58, 59].

The GB/SA formalism can be seamlessly integrated with our protocol for interaction energy calculation. The Hawkins-Cramer-Truhlar (HCT) model [60, 61] was used for calculating the effective Born radii. This model was shown to provide the closest agreement with explicit solvent simulations when calculating the dynamic properties of DNA [56]. While the van der Waals radii and screening constants required for the implicit solvent calculations involving amino acids were already available in GROMACS-4.5.5 [54], the atomic parameters of multiple nucleic acid atoms were missing. These were imported from the freely available source code of the TINKER 7.1 molecular modelling package (http://dasher.wustl.edu/tinker/). The interaction energies were then calculated using the Amber99SB-ILDN force field [51, 53] following the protocol described above. The calculations were performed in environments with relative permitivities of 4, 16, and 80. These values were chosen to approximate the electric properties of the protein interior, protein—nucleic acid interface, and the water environment, respectively.

It should be noted that the used GB/SA model places the dielectric medium everywhere around the molecular cavity, including the regions where neighbouring amino acids or base steps would be naturally present. It can be expected that this treatment affects the interaction energies. Future additions to the model could try to remedy this artificial behaviour by effectively including the cavitation and electrostatic effects of the neighboring residues.

## Results and Discussion

### Theoretical treatment of specificity

#### Interaction energy profiles

**Fig 3:**
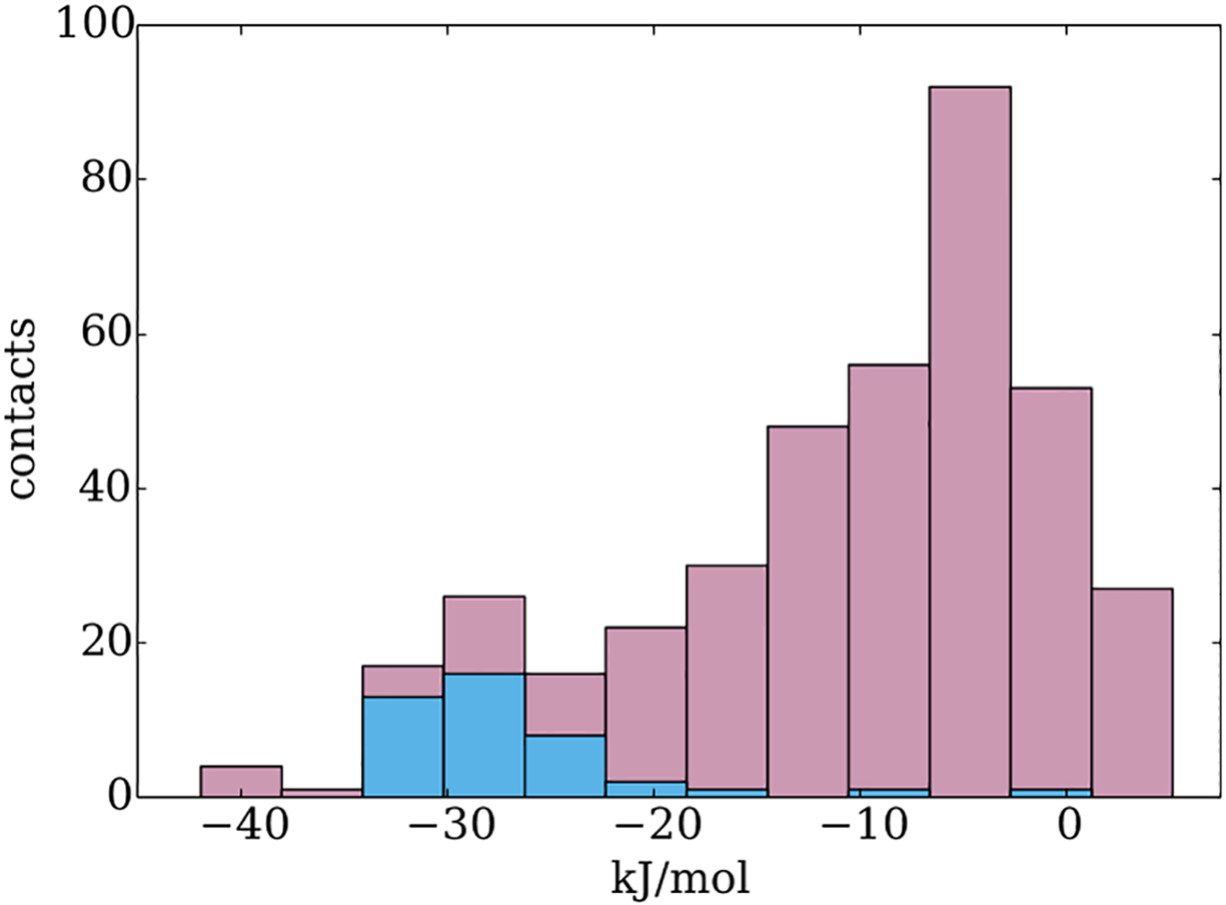
Interaction energy profile example: dAMP—asparagine dimers. The pink bars display the interaction energies of the entire distribution; the blue bars show the interaction energies provided by the members of its most stabilising cluster. The latter are shown in dark blue in Fig 2. The interaction energies were calculated in an environment with dielectric constant *ε* = 4.

To establish a link between the relative interaction energies of the various binding motifs and their geometrical conservation revealed by the clustering, we first constructed an interaction energy profile for each distribution. To this end, a histogram of the interaction energies provided by the dimers found in the distribution was created, and the number of bins was calculated from the Freedman-Diaconis formula [62]. The cluster containing the dimers providing on average the lowest (*i.e.*, most stabilising) interaction energies was then identified. A histogram of interaction energies provided by the members of this cluster was made, respecting the bin boundaries calculated for the respective distribution. The two histograms were then overlaid as shown in Fig 3.

#### Criteria of specificity

The clusters in each distribution represent geometrically conserved arrangements of the interacting partners. This conservation does not, however, automatically imply a role in the direct DNA sequence recognition. For example, contacts featuring single hydrogen bond donor or acceptor groups are naturally sterically constrained because of the directional requirements of hydrogen bonds and are therefore prone to being found in clusters. However, single hydrogen bonds are not sufficient to distinguish between individual DNA bases [12].

Based solely on geometrical criteria, the possibilities of specific base recognition by a single amino acid are limited to the few dimers featuring bidentate hydrogen bonds (see Introduction). Given that it is possible, especially in a high-dielectric environment, to achieve a similar level of stabilisation by utilising a combination of other non-covalent interaction modes [39], it may be desirable to augment this definition of specificity by explicitly considering the interaction energies of the respective dimer conformations.

We have already suggested that the presented spatial distributions effectively reflect the limited accessibility of the nucleotide in the DNA double helix to the approaching amino acids. Assuming that these distributions are configurationally saturated (in the same sense that an ergodic MD simulation would saturate the conformational space), the following requirements can be made for an interaction to be viewed as significant for the sequence recognition process:

1. The orientation of the amino acid relative to the DNA base (dNMP) must be found within one of the geometrical clusters. This condition implies that the respective interaction mode is utilised by many protein—DNA complexes and as such is not bound to be functional only under the unique local environment of a single protein family.
2. The cluster to which the dimer belongs must correspond to an attractive and most energetically favourable arrangement of the two partners.
3. Little to no other contacts other than those belonging to the distinct low-lying cluster are to be present within its interaction energy range. This criterion has two consequences. First, it enables identification of specificity-determining dimer geometries based on the respective interaction energies. Second, it implies that all dimers within that particular interaction energy range are highly sterically specific, as they could have been identified as forming a cluster.
4. The previous criteria specify energetically distinct geometries within the respective distributions. For an amino acid *A* to uniquely distinguish between individual DNA bases, the interaction energies found in dimers from the identified distinct low-lying cluster must also be lower (signed) than those provided by any contacts of *A* with any other base type. In other words, the stabilisation of the complex *A*—*B*, where *B* is the recognised DNA base, adopting a conformation falling to the distinct cluster, must be greater than the interaction energy found for any dimer of *A* with any other base type. This distinction is to be made for each of the nucleotide edges (Hoogsteen, Watson-Crick, sugar-phosphate) separately, as it may be possible for an amino acid to uniquely distinguish between different bases in each these regions.

Only when all these criteria are met can the coupling between the energetic and geometrical aspects of specificity be assumed. The interaction energy profile shown in Fig 3 already illustrates some of these distinctive characteristics; for a demonstration of the selectivity criteria involving all DNA bases, see S1–S4 Figs. The following sections cover the application of these rules to the various sets of amino acid—DNA base (dNMP) dimers and the identification of the distinct low-lying clusters described above.

### Observed binding preferences

#### Contacts with DNA bases

The simplest set to be analysed consists of only those side chain—DNA base dimers that feature a direct interaction between the two residues. The elimination of the charged sugar-phosphate group, for now, restricts the search space to those contacts in which its contribution is not essential for stabilisation of the interaction. Table 3 presents interactions that meet the above described criteria of specificity. These contacts were identified by visual inspection of the interaction energy profiles in the most numerous set constructed after identical protein—DNA complexes had been removed. The dimers that meet the criteria without exception are shown in bold; the other contacts appear significant, but some ambiguity in complying with the rules remains (for example, few non-clustering dimers provide similar interaction energies).

**Table 3:** DNA base—amino acid dimer types that can contribute to direct recognition.

Rel. permittivity	1	4	80
**Adenine**	**N^H^**,**Q^H^**,**K^s^**,**T^s^**	**N^H^**,**Q^H^**,**K^s^**,**T^s^**	**N^H^**,**Q^H^**,**K^s^**,**T^s^**
**Guanine**	**R^H^**,D^W^	**R^H^**,**D^W^**	**R^H^**,**D^W^**
**Cytosine**			
**Thymine**		**T^H^**	**T^H^**

The results for different dielectric environments are shown. Only the complexes in which the amino acid is in direct contact with the base moiety were considered. The dimer types for which an exceptionally good agreement between the interaction energy profile characterics and the criteria of specificity is observed are shown in bold. The superscript shows which edge of the DNA base (nucleotide) is contacted by the amino acid: ^H^—Hoogsteen edge; ^s^—sugar edge, ^P^—phosphate group, ^dis^—dispersion (stacking) interaction with the DNA base.

This set of side chain—DNA base dimers should predominantly be viewed as a control group, as it contains all the structural data necessary to recognise the contacts traditionally thought of as being involved in the direct readout. Indeed, the adenine—asparagine, adenine—glutamine, and guanine—arginine motifs were successfully recognised as forming specific contacts in all environments, supporting our hypothesis that the specificity can be observed by coupling the energetic and geometric features. The interaction energy profiles of these canonical amino acid—DNA base dimers can be seen in S5–S7 Figs. In addition, there were several other dimer types (adenine—lysine, adenine—threonine, guanine—aspartate, and thymine—threonine) with interaction energy profiles that shared the distinctive characteristics described above. Given that the properties of isolated DNA bases can be quite different from those of their nucleotide forms, these contacts will be investigated in greater detail if shown to be significant when the complete DNA residues are considered (see below). It should be noted that some of these dimers were already identified and explored in our previous work on DNA base interactions in the gas phase [41].

#### Base-directed contacts with dNMPs

We next investigated how addition of the sugar-phosphate group changes the binding preferences of the residues. Only dimers in which the side chain is in contact with the DNA base moiety were still considered. Table 4 shows the dNMP—amino acids dimer types that meet the stated criteria of specificity. As before, these analyses were performed on the set of protein—DNA complex structures obtained after discarding identical entries.

**Table 4:** dNMP—amino acid dimer types that can contribute to direct recognition.

Rel. permittivity	1	4	80
**dAMP**	**N^H^**,Q^H^	N^H^,**Q^H^**,K^s^,T^s^	N^H^,Q^H^,**K^s^**,T^s^
**dGMP**	R^H^	R^H^,D^W^	**R^H^**,**D^W^**,L^dis^
**dCMP**	**I^dis^**	I^dis^,K^s^	K^s^
**TMP**	S^H^,T^H^,**Y^H^**	T^H^,**Y^H^**	H^dis^,**T** ^H^,**Y^H^**

The results for different dielectric environments are shown. Contacts featuring interactions only with the sugar-phosphate backbone were excluded. Significant interactions not present in Table 3 are underlined. The dimer types for which an exceptionally good agreement between the interaction energy profile characterics and the criteria of specificity is observed are shown in bold. The superscript shows which edge of the DNA base (nucleotide) is contacted by the amino acid: ^H^—Hoogsteen edge; ^s^—sugar edge, ^P^—phosphate group, ^dis^—dispersion (stacking) interaction with the DNA base.

The specific recognition of adenine by asparagine or glutamine features a bidentate hydrogen bond between the side chain amide group of the amino acid and the C6 amino group/N7 atoms of the base. These canonical interactions remain the most energetically favourable even in the presence of the sugar-phosphate group, as long as the low dielectric constant (*ε* = 1 or 4) of the local environment is assumed. Despite meeting all of our specificity requirements, the interaction of adenine with threonine was realised in too few contacts to allow deeper statistical investigation. This interaction is realised in the minor groove *via* a single hydrogen bond between the side chain hydroxyl group of the amino acid and the N3 atom of the base.

When a water-like (*ε* = 80) dielectric environment is assumed, the interaction between adenine and lysine is the only one to display energetically and geometrically distinctive characteristics. This interaction features a single hydrogen bond between the terminal amino group of the amino acid and the N3 atom of the base. In addition, a set of van der Waals contacts is present between the aliphatic lysine side chain and the 2’-deoxyribose atoms. It should be noted that the members of a single cluster in the distribution of cytosine—lysine dimers adopt an analogous geometry (Tables 4 and 5). However, these latter contacts form a significant cluster only when the most redundant set of protein—DNA complexes is considered, and the respective binding motif is thus not widely utilised.

**Table 5:** dNMP—amino acid dimer types that can contribute to direct recognition.

Rel. permittivity	1	4	80
**dAMP**	**N^H^**,**Q^H^**,T^s^	**N^H^**,**Q^H^**,K^s^	N^H^,**Q^H^**,**K^s^**,T^s^
**dGMP**		**R^H^**,**D^W^**,L^dis^	**R^H^**,**D^W^**,L^dis^
**dCMP**	**Q^P^**,**I^dis^**	**Q^P^**,**I^dis^**	**Q^P^**,K^s^
**TMP**	**Q^P^**,S^H^,T^H^,**Y^H^**	**Q^P^**,S^H^,T^H^,**Y^H^**	**Q^P^**,S^H^,T^H^

The results for different dielectric environments are shown. Contacts featuring interactions only with the sugar-phosphate backbone were included. Significant interactions not present in Table 4 are underlined. The dimer types for which an exceptionally good agreement between the interaction energy profile characterics and the criteria of specificity is observed are shown in bold. The superscript shows which edge of the DNA base (nucleotide) is contacted by the amino acid: ^H^—Hoogsteen edge; ^s^—sugar edge, ^P^—phosphate group, ^dis^—dispersion (stacking) interaction with the DNA base.

The previously identified clusters in the distributions of asparagine, glutamine, and threonine lost some of their distinctive characteristics with the increased dielectric constant. Notably, several non-cluster contacts began to offer the same interaction energies as those found in the clusters. Thus, we hypothesise that the specific interaction with the charged lysine plays a role in recognition of adenine over a larger distance, while the nucleic acid remains enveloped by a hydration shell. When the electric properties of the interfacial region are described by a high dielectric constant, interactions with amino acids other than lysine do not provide similar levels of base-specific resolution. The increased relative selectivity in a water-like environment compared to the low dielectric was also observed in other motifs involving charged amino acids (see below).

**Fig 4:**
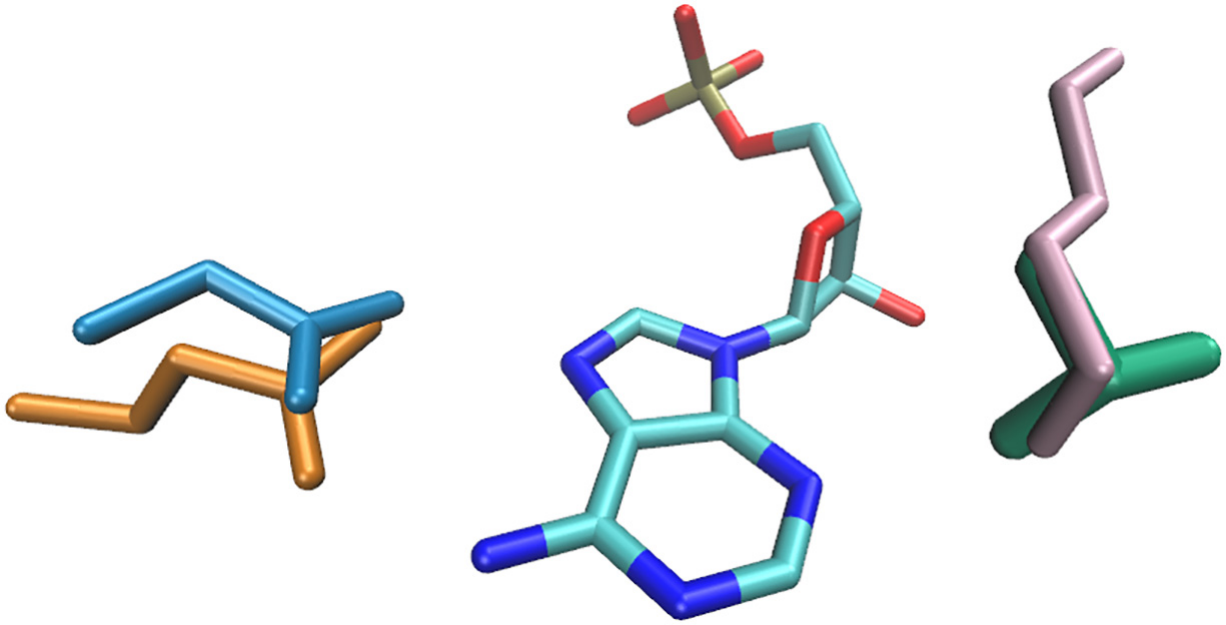
Recognition of adenine by asparagine (blue), glutamine (orange), lysine (pink), and threonine (green).

The atomic-level details of the specific interactions involving adenine are shown in Fig 4. The interaction energy profiles of the abovementioned dimers can be seen in S9–S12 Figs.

The canonical recognition of guanine by arginine is realised *via* a bidentate hydrogen bond between the terminal guanidino group of the amino acid and the O6 and N7 atoms of the base. This interaction also displays the distinctive characteristics only when the solvent effects are taken into account. Partial screening of the atomic charges is needed for the recognition in this case because the fine details of the interaction with the base are otherwise lost due to the dominant electrostatic attraction of the amino acid with the DNA backbone.

The specific interaction of aspartate with guanine features a bidentate hydrogen bond between the terminal carboxylic group of the amino acid and the N1 and N2 amino group atoms of the base. This binding obviously interferes with the Watson-Crick pairing between DNA bases. A deeper analysis of the complexes from which this contact originates (PDB IDs 1JB7, 1OMH, 1PO6, 1XJV, and 3ZH2) reveals that the motif is utilised in the recognition of aptameric, telomeric, or otherwise strained DNA structures. While likely not involved in routine sequence recognition, this highly stabilising interaction contributes to and can even be crucial for the recognition of non-canonical forms of DNA. This exceptional case illustrates the robustness of our general criteria of specificity. Without any prior information about the structures present in the set, we were able to find a group of non-homologous proteins which, nonetheless, featured the same binding motif involved in the sequence recognition in the respective complexes. Interestingly, the interactions of aspartate and glutamate with guanine *via* the Watson-Crick edge were found to provide to the most favourable binding free energies of all amino acid—DNA base dimer types [39].

**Fig 5:**
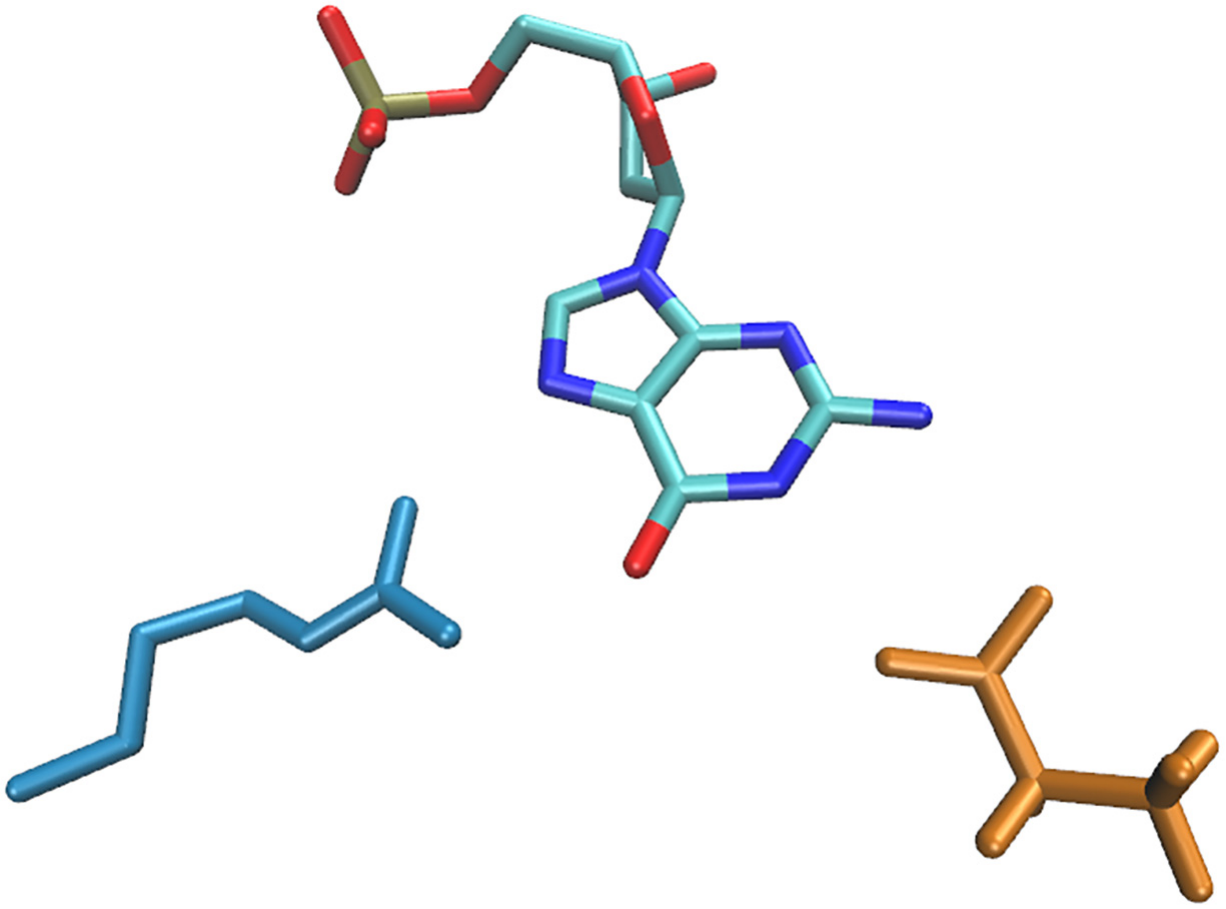
Recognition of guanine by arginine (blue) and aspartate (orange).

The atomic-level details of the specific interactions involving guanine are shown in Fig 5. The interaction energy profiles of the two dimers can be seen in S13 and S14 Figs.

The possible recognition of guanine by asparagine or glutamine through the sugar edge did not display the clustering characteristics. Similarly, there were no distinct interactions involving cytosine as a base, especially when the solvent effects were taken into account and the more restrictive sequence identity criteria were applied. In our previous work, we were able to identify distinct clusters of asparagine and tyrosine side chains forming contacts with cytosine *via* a single hydrogen bond featuring the O2 atom of the DNA base as an acceptor [41]. The low population of these clusters (6 and 4 contacts for asparagine and tyrosine dimers, respectively) meant that the removal of only few protein—DNA complexes from the data set due to redundancy put the population these motifs below the threshold needed for the detection of the clusters. As we have now taken into consideration the presence of the clusters across various redundancy levels when identifying the distinct interaction motifs, these contacts do not appear in Table 3. It is, however, possible that, given a larger data set, the significance of these motifs could become apparent. The absence of the motifs involving guanine identified in ref. [41] follows the same reasoning.

**Fig 6:**
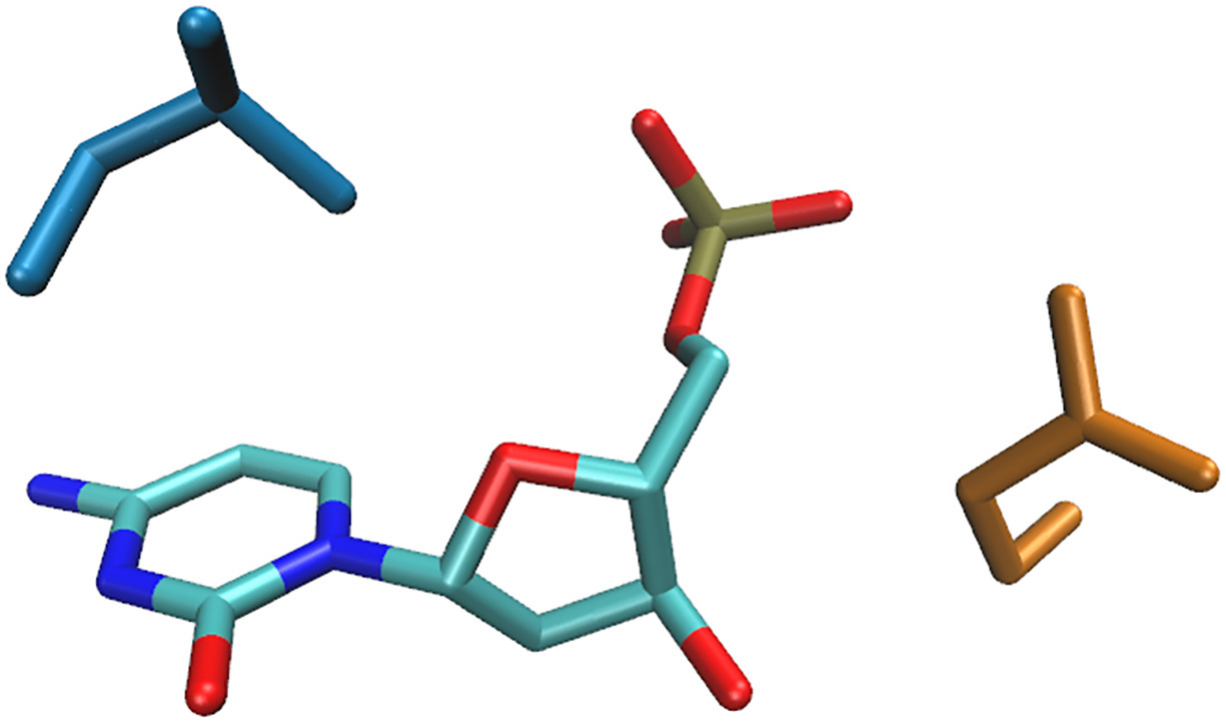
Interactions of cytosine with isoleucine (blue) and glutamine (orange).

A cluster of isoleucine that displays preference towards cytosine *in vacuo* consists of contacts featuring van der Waals interactions involving almost all atoms of the amino acid side chain and almost all atoms of the nucleotide (Fig 6; the corresponding interaction energy profile is shown in S15 Fig). We found that the dGMP—leucine dimers adopt a similar geometry. However, the most energetically favourable cluster lacks some of the distinctive characteristics in this case. Although it is known that specific hydrophobic amino acids are crucial for the stabilisation of some repressor/operator complexes [14], there has not been any sign of a universal one-to-one correspondence between the interacting residues. It is, of course, possible that the current treatment of the solvent effects is inadequate for the complete description of the stabilisation provided by this binding motif.

The distinctive characteristics of the interactions of serine, threonine, and tyrosine with thymine become prominent only after the addition of the sugar-phosphate group to the DNA base. All of these binding motifs involve a hydrogen bond between the donor hydroxyl group of the amino acid side chain and one of the phosphate group acceptor oxygen atoms. The C5 methyl group of thymine sterically stabilises these motifs by interacting with the hydroxyl group oxygen atom from the opposite side. This interaction is not possible in contacts with the other DNA bases. In addition, the hydrophobic effect may stabilise the interaction of the two methyl groups in contacts involving threonine. This aditional stabilisation may be the cause of the higher population and stereospecificity of the motif involving threonine compared to that containing serine.

**Fig 7:**
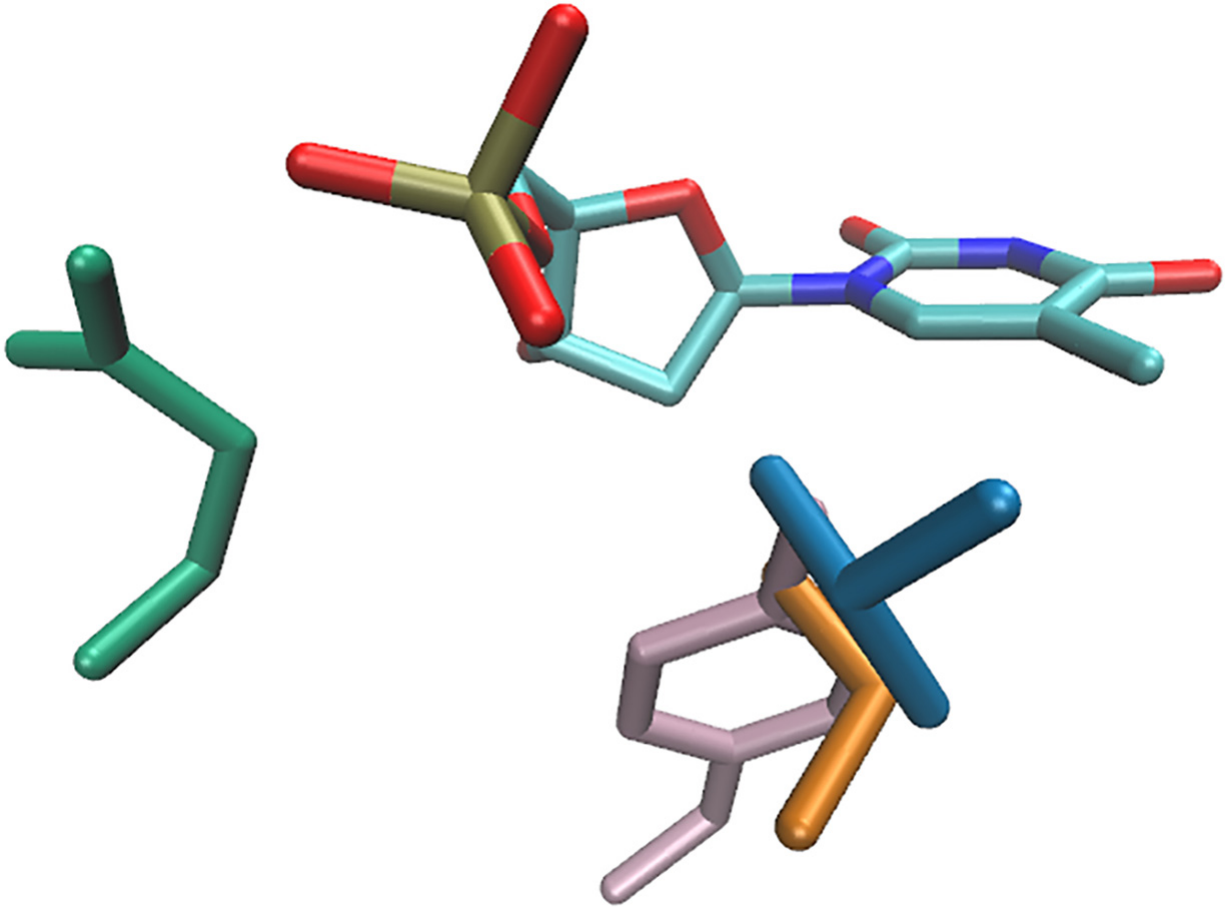
Recognition of thymine by threonine (blue), serine (orange), tyrosine (pink), and glutamine (green).

The atomic-level details of the specific interactions involving thymine are shown in Fig 7. The interaction energy profiles of the abovementioned dimer types can be seen in S16–S18 Figs.

#### DNA backbone-directed contacts

Finally, we considered distributions involving all amino acid—dNMP dimers, including those featuring solely contacts with the DNA backbone. Table 5 presents those dimer types in which the amino acid side chains form clusters displaying the distinct properties described above. These preferences were found on the set from which identical protein entries had been discarded.

In addition to the previously identified dimers, two notable new interactions appeared. The contacts of cytosine and thymine with glutamine feature a single hydrogen bond between the side chain amide group of the amino acid and one of the phosphate group oxygen atoms. No interaction with the nitrogenous base moieties are present. These contacts display the distinct characteristics *in vacuo* as well as in the tested dielectrics. We are unsure why the amino acids prefer the pyrimidine-containing nucleotides. Similar interactions involving the purine nucleotides are not present in this set. This interaction motif can be observed for the pyrimidine bases even after applying the most strict redundancy-culling criteria. It is possible that the apparent preference displayed by these dimers is the result of an inadequate sampling of the configurational space realised in the currently available structures of protein—DNA complexes (see below).

The interaction energy profiles of these dimers can be seen in S19 and S20 Figs. Atomic-level details of the interactions are shown in Figs 6 and 7 for cytosine and thymine, respectively.

### Possible extensions of the model

#### Structural data

A clear drawback of our interaction energy profile-based specificity definition is that only sufficiently represented motifs will be detected. If this analysis is to be considered complete, we must assume that all amino acid preferences for DNA bases can already be detected in the binding modes realised in the currently available structures of protein—DNA complexes. It is unfortunate that the number of amino acid—DNA residue dimers is still an order of magnitude lower compared to the number of amino acid—amino acid contacts available from high-quality protein structures [63]. This insufficiency is most apparent when using more strict homology-reducing criteria. While most of the motifs presented here can still be found in these less redundant sets, reduction of their population by a half (or more) often makes the presented interaction energy profile-based technique inappropriate due to the insufficient resolution of the histograms.

#### Water-mediated interactions

While the treatment of the solvent as a continuous dielectric offers a significant improvement over the gas phase calculations, it is inappropriate for the treatment of interactions that naturally involve a bridging water molecule. Previous work found that almost one fifth of all contacts involve a solvent-mediated interaction, most of them directed at the DNA backbone [27]. The crucial role of well-ordered water molecules in sequence recognition has been described in the *trp* repressor/operator complex [14]. Unfortunately, large variations in the number of water molecules present in the crystallographic structures exist in the range of resolutions used in this study. For example, an increase in resolution from 2.6 Å to 1.9 Å for the nucleosome core particle has revealed over 2,500 more water molecules [4]. Therefore, even if the solvent molecules that can be found in the protein—DNA structures used in this work were included in the calculations, it is be likely that some natural water-mediated interactions would still be missing.

#### Calculation of additional binding free energy components

The presented approach to calculating the potential energy of the interaction between two residues provides, of course, a limited approximation of the binding free energy, which is the biophysically relevant potential. In particular, no explicit treatment of the entropic effects was attempted, although a part of the solvent entropy could have effectively been captured by the cavitation component of the GB/SA approach. While the entropy of the solutes could be estimated from normal mode vibrational analysis or molecular dynamics trajectory [64], the determination of this term is beyond the scope of this paper.

Free energy difference calculations with explicit representation of the solvent molecules, such as those recently performed [39], are, of course, the most appropriate computational approach to fully accounting for all components of the binding free energy. The omission of the explicit treatment of the solvent entropy in our paper could have resulted in some motifs, especially those involving non-polar amino acids, being significantly mistreated. In fact, very few of the herein identified distinct clusters involve interactions which do not feature a dominant electrostatic component. On the other hand, the interaction energies, and especially the interaction energy differences, of the identified dimers (which almost unanimously feature hydrogen bonds) can be expected to be reasonably close to the biophysically relevant energy values.

One possible validation of our approach would be the comparison of the calculated interaction energies with the free energy differences derived from statistical potentials obtained by evaluating the relative probabilities of the various interaction motifs. Protein—nucleic acid complexes were previously explored using this approach by Mandel-Gutfreund and Margalit [25] and others [65–67]; for a thorough list of references related to the study of protein structures see ref. [68]. Such an approach could, in principle, be used to correctly describe not only the motifs in which the enthalpic contribution to the free energy of binding is dominant (as can be assumed for the motifs described in this paper), but also the motifs in which binding is driven by hydrophobic and other entropic effects. This would require that the conformational space of all amino acid—nucleotide dimers be adequately sampled by the dimer geometries extracted from the currently available structures of protein—DNA complexes. Based on the populations of some of the motifs presented in this article, we think that this requirement could hardly be met as of now.

#### Interactions of larger residue blocks

As this study was focused on finding the binding preferences at the one-to-one correspondence level, contacts spanning multiple base steps or featuring interactions with both DNA residues in a base pair were not explicitly treated as such. It has been shown that the assumption of additivity of individual amino acid—mononucleotide interactions is a reasonable approximation in the search of DNA binding sites [69]. If the preferences towards oligonucleotide blocks were to be probed in as exhaustive a manner as done for individual DNA bases, it would become apparent that the number of contacts provided by the currently available protein—DNA structures would not be sufficient for a reasonable analysis. If one were to also consider the preferences of larger peptide blocks, the sheer number of possible sequence variations would quickly become greater than the number of available contacts altogether. It is, however, very well possible that the extension of the presented methodology to cover the interactions of these larger biomolecular fragments could reveal additional interaction motifs significant for the process of direct sequence readout. Alternatively, the energetics of interactions with neighbouring base steps or amino acids could, in some cases, disrupt the observed binding preferences. Explicit solvent MD simulations of selected oligopeptide/oligonucleotide complexes currently seem to be the best theoretical approach to investigate the binding preferences involving these larger fragments.

#### Role of non-specific contacts

Depending on the applied redundancy-culling criteria, only between one tenth and one quarter of all amino acid—DNA residue dimers were found in the clusters. It appears rational to ask what is the role of the remaining contacts. The only thing that can be said based on our study about these non-clustering dimers is that they do not massively participate in binding motifs involved in the direct readout of single DNA bases. On the other hand, given that the protein side of the interaction interface is occasionally limited to only several amino acids, there may be little space left for random noise. These remaining contacts can thus well serve as modulators of the recognized motifs involved in the direct readout, or, alternatively, be involved in facilitating the shape recognition or other, more complex phenomena. This can only be decided in the context of each individual DNA-binding protein.

#### Evolution of the protein—DNA interface

In this work, we have shown that several amino acid—DNA nucleotide combinations considerably extend the library of motifs that can be utilised in direct sequence recognition. The significance and conservation of these motifs across various protein families may have had unknown consequences on the evolution of transcription factors and their cognate DNA sequences. We have so far made very general predictions about the interactions between individual residues without probing the original biomacromolecules. Indeed, our next logical step will be to investigate the relationship between the utilisation of the specific motifs and the source protein structures.

## Supporting Information

S1 Fig Demonstration of the criteria of specificity: the interaction energy profile of the dAMP—glutamine dimer. The interaction energies were calculated in an environment with dielectric constant *ε* = 4. Only those dimers in which the amino acid interacts with the base moiety of the nucleotide were considered in the construction of the profile. No two 100% identical proteins were present in the set from which the dimers were extracted. The pink histograms show the interaction energy profile of the entire distributions; the blue histograms display the interaction energy profile of its most energetically stabilising cluster. Note how the cluster in this distribution meets the specificity criteria:
it represents the most favourable arrangement of the partners within the distribution,very few other (*i.e.*, non-cluster) contacts within the profile provide similar interaction energies as the cluster’s members,the interactions with the other DNA bases (S2–S4 Figs), do not contain a significant number of contacts with similar interaction energies.
(TIF)

S2 Fig Demonstration of the criteria of specificity: the interaction energy profile of the dCMP—glutamine dimer. The pink histograms show the interaction energy profile of the entire distributions; the blue histograms display the interaction energy profile of its most energetically stabilising cluster. Note how the character of the cluster (the shape and position of the cluster profile relative to the profile of the distribution) differs from that of the cluster in dAMP—glutamine distribution (S1 Fig). The selection of the data set for the construction of the profile and other computational details are the same as in S1 Fig.
(TIF)

S3 Fig Demonstration of the criteria of specificity: the interaction energy profile of the dGMP—glutamine dimer. The pink histograms show the interaction energy profile of the entire distributions; the blue histograms display the interaction energy profile of its most energetically stabilising cluster. Note how the character of the cluster (the shape and position of the cluster profile relative to the profile of the distribution) differs from that of the cluster in dAMP—glutamine distribution (S1 Fig). The selection of the data set for the construction of the profile and other computational details are the same as in S1 Fig.
(TIF)

S4 Fig Demonstration of the criteria of specificity: the interaction energy profile of the TMP—glutamine dimer. The pink histograms show the interaction energy profile of the entire distributions; the blue histograms display the interaction energy profile of its most energetically stabilising cluster. Note how the character of the cluster (the shape and position of the cluster profile relative to the profile of the distribution) differs from that of the cluster in dAMP—glutamine distribution (S1 Fig). The selection of the data set for the construction of the profile and other computational details are the same as in S1 Fig.
(TIF)

S5 Fig Interaction energy profile of a canonical amino acid—DNA base dimer involved in the direct readout: adenine—asparagine. The energetically lowest lying cluster (blue) shows distinctive characteristics, as defined in text and in S1 Fig legend. The interaction energies were calculated in an environment with dielectric constant *ε* = 4. Only those dimers in which the amino acid interacts with the base moiety were considered in the construction of the interaction energy profiles. No two 100% identical proteins were present in the set from which the dimers were extracted.
(TIF)

S6 Fig Interaction energy profile of a canonical amino acid—DNA base dimer involved in the direct readout: adenine—glutamine. The energetically lowest lying cluster (blue) shows distinctive characteristics, as defined in text and in S1 Fig legend. The selection of the data set for the construction of the profile and other computational details are the same as in S5 Fig.
(TIF)

S7 Fig Interaction energy profile of a canonical amino acid—DNA base dimer involved in the direct readout: guanine—arginine. The energetically lowest lying cluster (blue) shows distinctive characteristics, as defined above. The selection of the data set for the construction of the profile and other computational details are the same as in S5 Fig. The “envelope” of non-cluster contacts in the profile is caused by the symmetry of the arginine guanidino group: four energetically equivalent orientations of the side chain involving the guaninidino group as hydrogen bond donor exist; however, the cluster consists of only one of those. One of these alternative orientations is shown in S8 Fig.
(TIF)

S8 Fig dGMP—arginine dimer: one of four energetically equivalent geometries. These geometries contribute to the “envelope” of non-cluster contacts covering the cluster profile (blue) in S7 Fig. Compare with Fig 5 (blue) in the main text.
(TIF)

S9 Fig Interaction energy profile of a dimer involving dAMP in which the energetically lowest lying cluster displays some of the distinctive characteristics: dAMP—asparagine. The interaction energies were calculated in an environment with dielectric constant *ε* = 4. Only those dimers in which the amino acid interacts with the base moiety of the nucleotide were considered in the construction of the interaction energy profile. No two 100% identical proteins were present in the set from which the dimers were extracted.
(TIF)

S10 Fig Interaction energy profile of a dimer involving dAMP in which the energetically lowest lying cluster displays some of the distinctive characteristics: dAMP—glutamine. The selection of the data set for the construction of the profile and other computational details are the same as in S9 Fig.
(TIF)

S11 Fig Interaction energy profile of a dimer involving dAMP in which the energetically lowest lying cluster displays some of the distinctive characteristics: dAMP—threonine. The selection of the data set for the construction of the profile and other computational details are the same as in S9 Fig.
(TIF)

S12 Fig Interaction energy profile of a dimer involving dAMP in which the energetically lowest lying cluster displays some of the distinctive characteristics: dAMP—lysine. The interaction energies were calculated in an environment with dielectric constant *ε* = 80. The selection of the data set for the construction of the profile is the same as in S9 Fig.
(TIF)

S13 Fig Interaction energy profile of a dimer involving dGMP in which the energetically lowest lying cluster displays some of the distinctive characteristics: dGMP—arginine. The interaction energies were calculated in an environment with dielectric constant *ε* = 80. Only those dimers in which the amino acid interacts with the base moiety of the nucleotide were considered in the construction of the interaction energy profile. No two 100% identical proteins were present in the set from which the dimers were extracted. The “envelope” of isoenergetic non-cluster contacts covering the cluster profile is present for the symmetry reasons discussed in the legend of S7 Fig and illustrated in S8 Fig.
(TIF)

S14 Fig Interaction energy profile of a dimer involving dGMP in which the energetically lowest lying cluster displays some of the distinctive characteristics: dGMP—aspartate. The selection of the data set for the construction of the profile and other computational details are the same as in S13 Fig.
(TIF)

S15 Fig Interaction energy profile of the dimer involving dCMP in which the energetically lowest lying cluster displays some of the distinctive characteristics: dCMP—isoleucine. The interaction energies were calculated in an environment with dielectric constant *ε* = 1. Only those dimers in which the amino acid interacts with the base moiety of the nucleotide were considered in the construction of the interaction energy profile. No two 100% identical proteins were present in the set from which the dimers were extracted.
(TIF)

S16 Fig Interaction energy profile of a dimer involving TMP in which the energetically lowest lying cluster displays some of the distinctive characteristics: TMP—serine. The interaction energies were calculated in an environment with dielectric constant *ε* = 4. All amino acid—nucleotide dimers were considered in the construction of the interaction energy profile. No two 100% identical proteins were present in the set from which the dimers were extracted.
(TIF)

S17 Fig Interaction energy profile of a dimer involving TMP in which the energetically lowest lying cluster displays some of the distinctive characteristics: TMP—threonine. The selection of the data set for the construction of the profile is the same as in S16 Fig.
(TIF)

S18 Fig Interaction energy profile of a dimer involving TMP in which the energetically lowest lying cluster displays some of the distinctive characteristics: TMP—tyrosine. The selection of the data set for the construction of the profile is the same as in S16 Fig.
(TIF)

S19 Fig Interaction energy profile of a dimer in which the energetically lowest lying cluster displays some of the distinctive characteristics: dCMP—glutamine. The binding motif realised in the distinctive cluster features only an interaction with the DNA backbone. The interaction energies were calculated in an environment with dielectric constant *ε* = 4. All amino acid—nucleotide dimers were considered in the construction of the interaction energy profile. No two 100% identical proteins were present in the set from which the dimers were extracted.
(TIF)

S20 Fig Interaction energy profile of a dimer in which the energetically lowest lying cluster displays some of the distinctive characteristics: TMP—glutamine. The binding motif realised in the distinctive cluster features only an interaction with the DNA backbone. The selection of the data set for the construction of the profile is the same as in S19 Fig.
(TIF)
