# Supplementary figures and images for: Sequence-Specific Recognition of DNA by Proteins: Binding Motifs Discovered Using a Novel Statistical/Computational Analysis
Source: PLoS One. 2016 Jul 6;11(7):e0158704. doi: 10.1371/journal.pone.0158704 (PMC4934765; doi:10.1371/journal.pone.0158704)

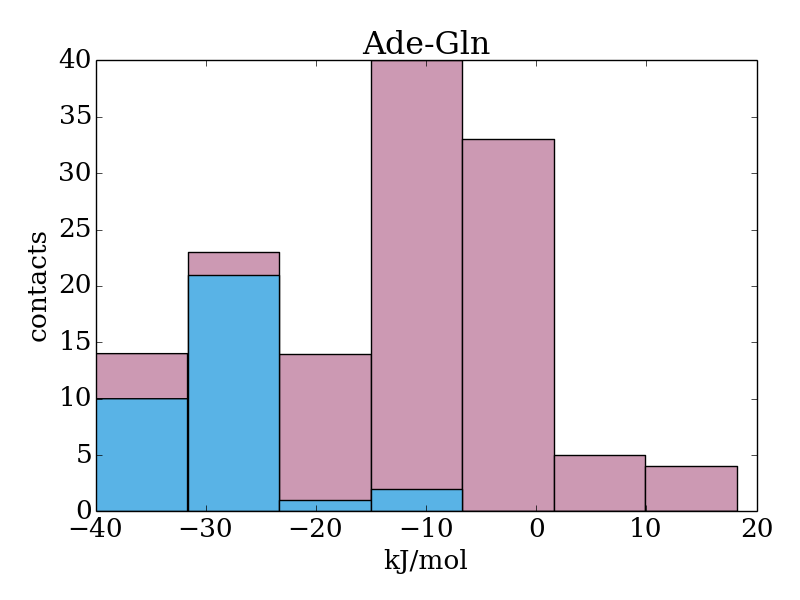

Supplement: S1 Fig — The interaction energies were calculated in an environment with dielectric constant ε = 4. Only those dimers in which the amino acid interacts with the base moiety of the nucleotide were considered in the construction of the profile. No two 100% identical proteins were present in the set from which the dimers were extracted. The pink histograms show the interaction energy profile of the entire distributions; the blue histograms display the interaction energy profile of its most energetically stabilising cluster. Note how the cluster in this distribution meets the specificity criteria: it represents the most favourable arrangement of the partners within the distribution,very few other (i.e., non-cluster) contacts within the profile provide similar interaction energies as the cluster’s members,the interactions with the other DNA bases (S2–S4 Figs), do not contain a significant number of contacts with similar interaction energies. (TIF) [file pone.0158704.s001.tif]

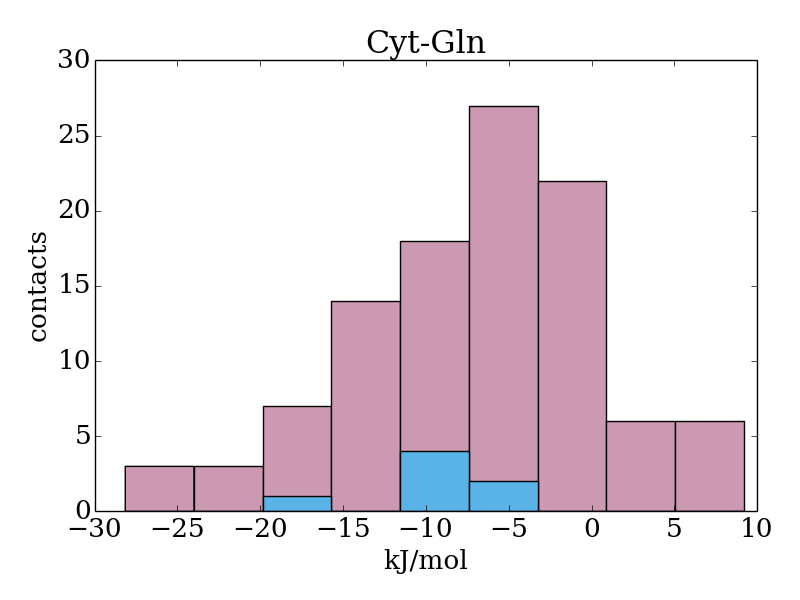

Supplement: S2 Fig — The pink histograms show the interaction energy profile of the entire distributions; the blue histograms display the interaction energy profile of its most energetically stabilising cluster. Note how the character of the cluster (the shape and position of the cluster profile relative to the profile of the distribution) differs from that of the cluster in dAMP—glutamine distribution (S1 Fig). The selection of the data set for the construction of the profile and other computational details are the same as in S1 Fig. (TIF) [file pone.0158704.s002.tif]

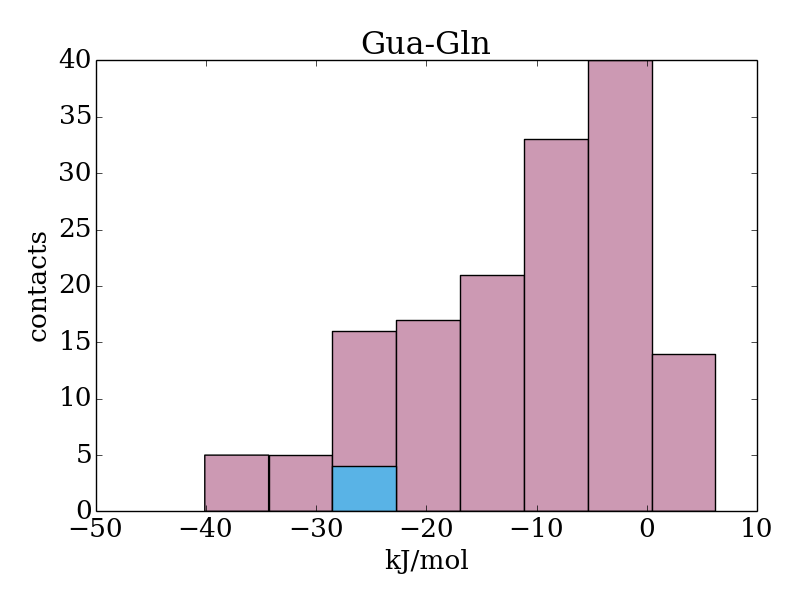

Supplement: S3 Fig — The pink histograms show the interaction energy profile of the entire distributions; the blue histograms display the interaction energy profile of its most energetically stabilising cluster. Note how the character of the cluster (the shape and position of the cluster profile relative to the profile of the distribution) differs from that of the cluster in dAMP—glutamine distribution (S1 Fig). The selection of the data set for the construction of the profile and other computational details are the same as in S1 Fig. (TIF) [file pone.0158704.s003.tif]

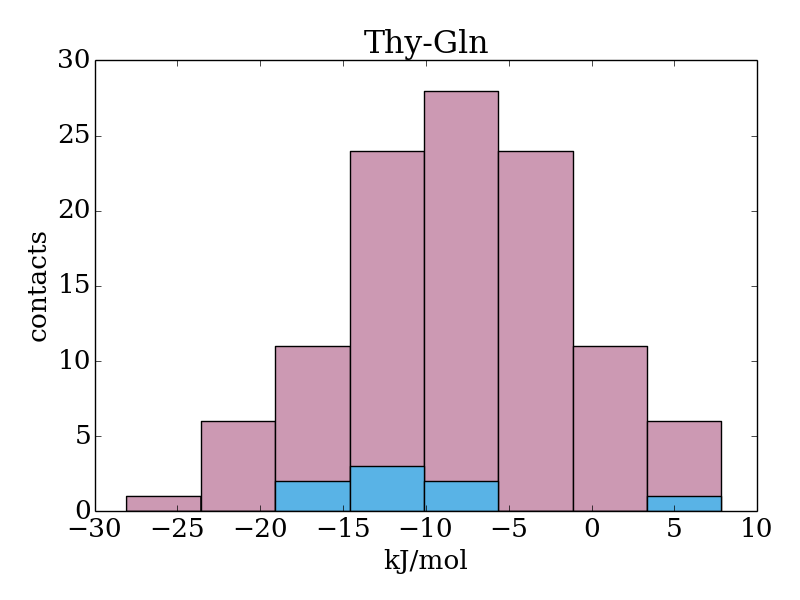

Supplement: S4 Fig — The pink histograms show the interaction energy profile of the entire distributions; the blue histograms display the interaction energy profile of its most energetically stabilising cluster. Note how the character of the cluster (the shape and position of the cluster profile relative to the profile of the distribution) differs from that of the cluster in dAMP—glutamine distribution (S1 Fig). The selection of the data set for the construction of the profile and other computational details are the same as in S1 Fig. (TIF) [file pone.0158704.s004.tif]

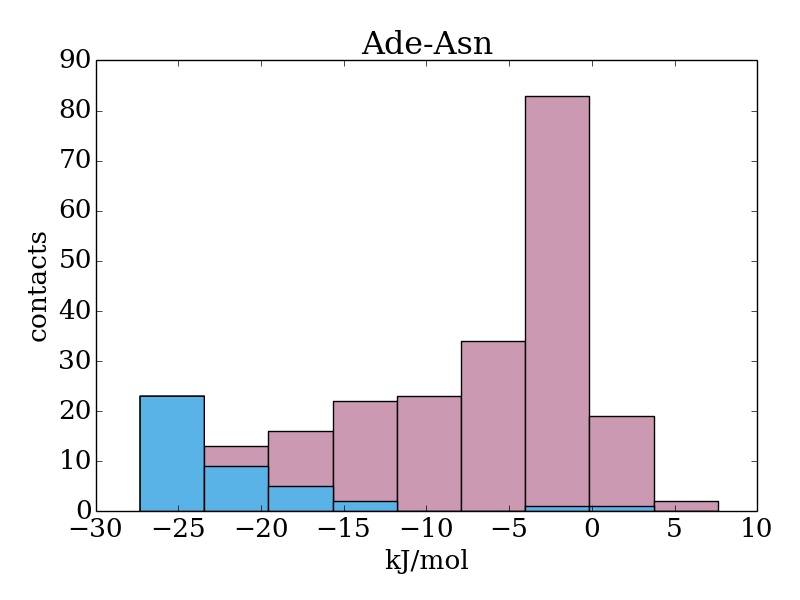

Supplement: S5 Fig — The energetically lowest lying cluster (blue) shows distinctive characteristics, as defined in text and in S1 Fig legend. The interaction energies were calculated in an environment with dielectric constant ε = 4. Only those dimers in which the amino acid interacts with the base moiety were considered in the construction of the interaction energy profiles. No two 100% identical proteins were present in the set from which the dimers were extracted. (TIF) [file pone.0158704.s005.tif]

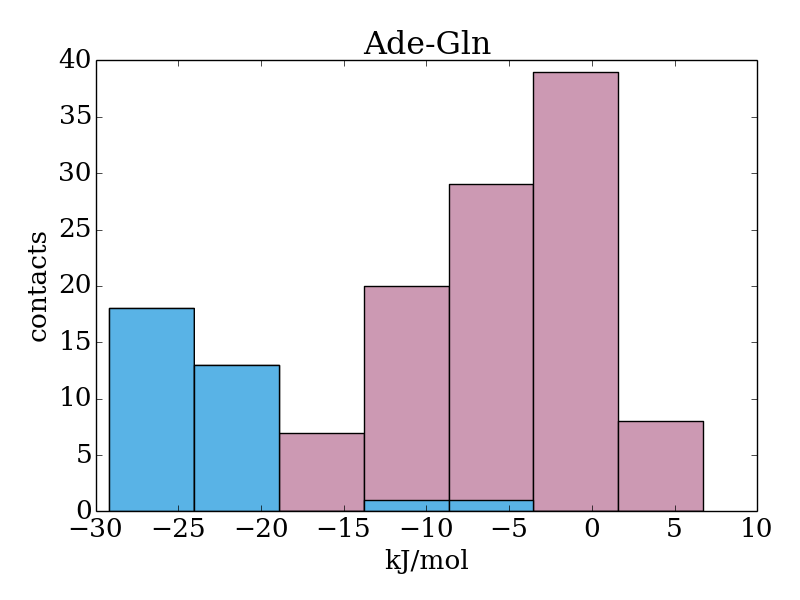

Supplement: S6 Fig — The energetically lowest lying cluster (blue) shows distinctive characteristics, as defined in text and in S1 Fig legend. The selection of the data set for the construction of the profile and other computational details are the same as in S5 Fig. (TIF) [file pone.0158704.s006.tif]

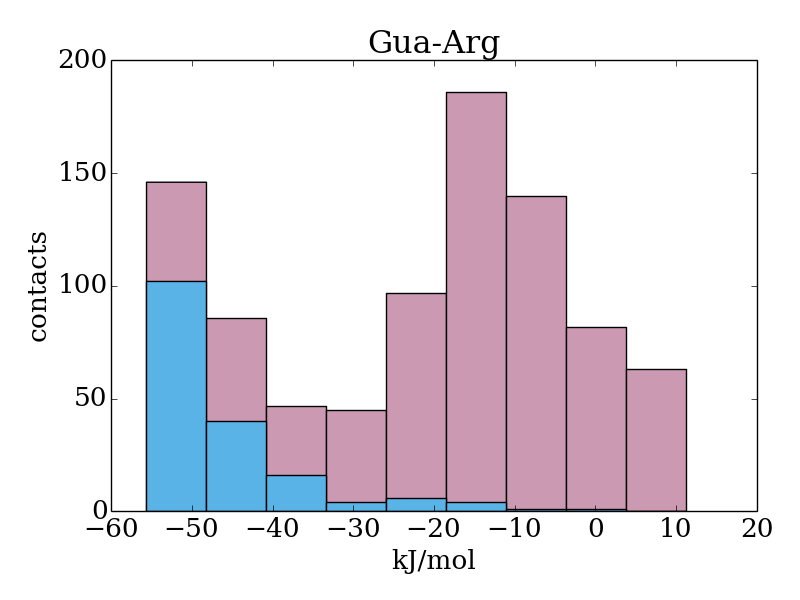

Supplement: S7 Fig — The energetically lowest lying cluster (blue) shows distinctive characteristics, as defined above. The selection of the data set for the construction of the profile and other computational details are the same as in S5 Fig. The “envelope” of non-cluster contacts in the profile is caused by the symmetry of the arginine guanidino group: four energetically equivalent orientations of the side chain involving the guaninidino group as hydrogen bond donor exist; however, the cluster consists of only one of those. One of these alternative orientations is shown in S8 Fig. (TIF) [file pone.0158704.s007.tif]

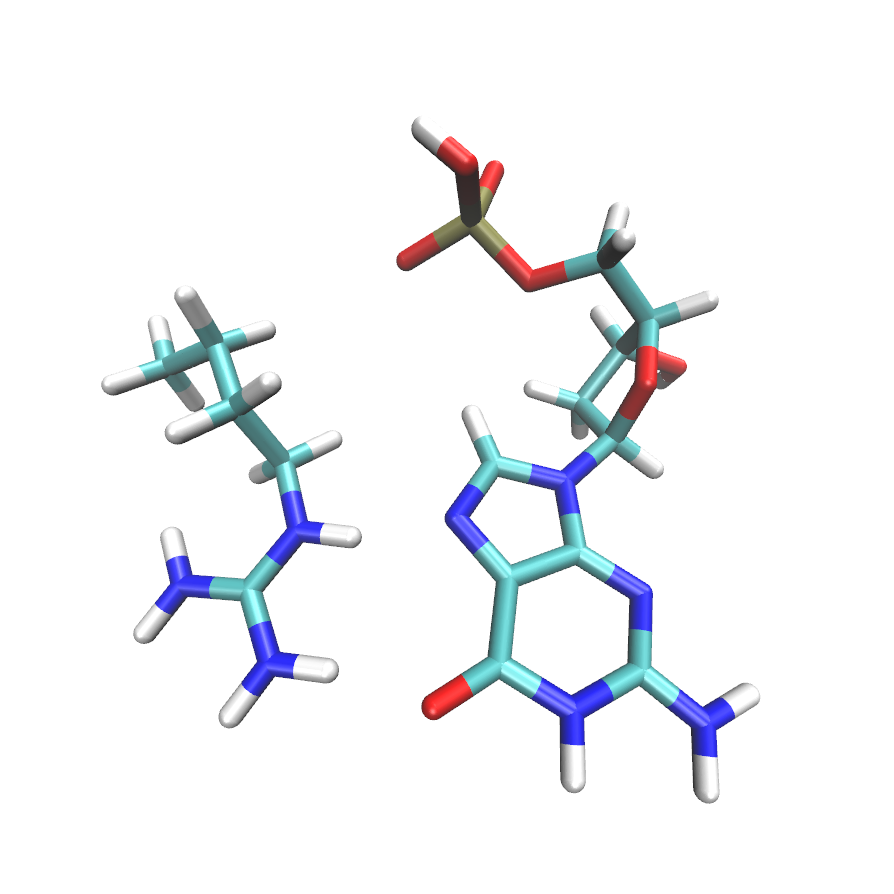

Supplement: S8 Fig — These geometries contribute to the “envelope” of non-cluster contacts covering the cluster profile (blue) in S7 Fig. Compare with Fig 5 (blue) in the main text. (TIF) [file pone.0158704.s008.tif]

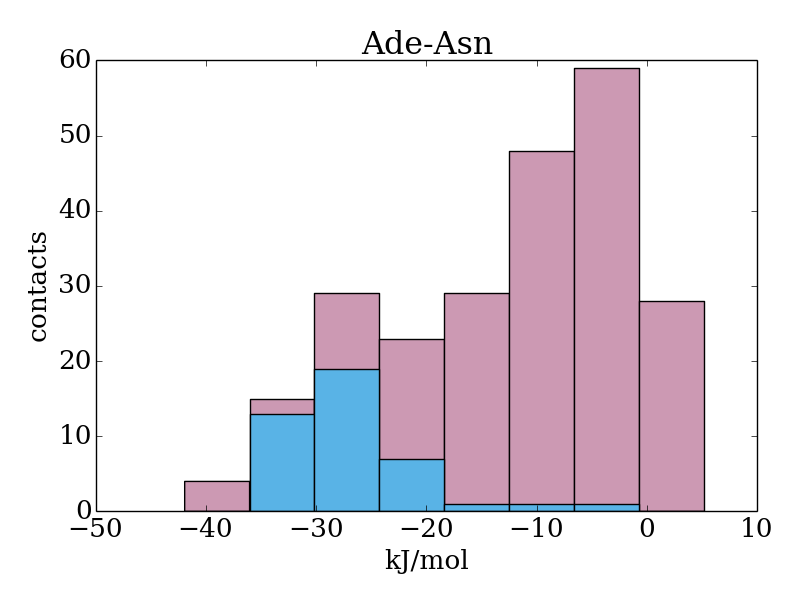

Supplement: S9 Fig — The interaction energies were calculated in an environment with dielectric constant ε = 4. Only those dimers in which the amino acid interacts with the base moiety of the nucleotide were considered in the construction of the interaction energy profile. No two 100% identical proteins were present in the set from which the dimers were extracted. (TIF) [file pone.0158704.s009.tif]

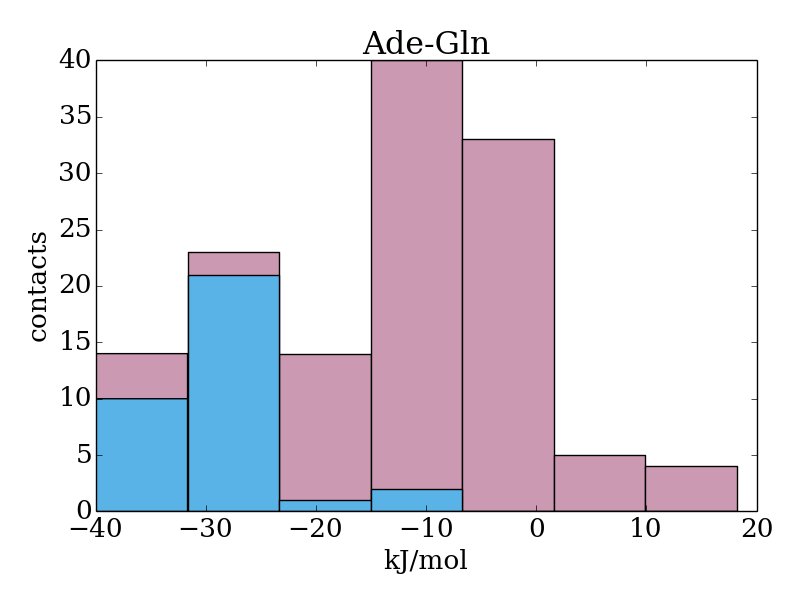

Supplement: S10 Fig — The selection of the data set for the construction of the profile and other computational details are the same as in S9 Fig. (TIF) [file pone.0158704.s010.tif]

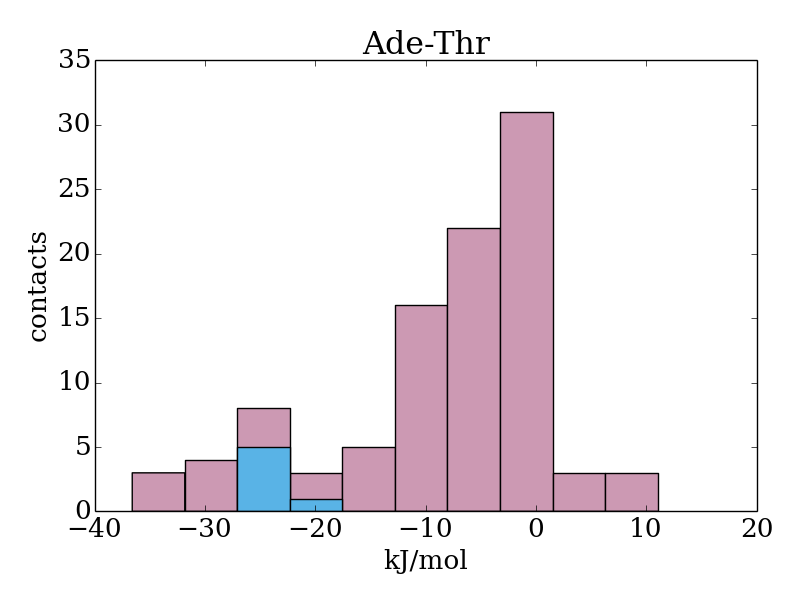

Supplement: S11 Fig — The selection of the data set for the construction of the profile and other computational details are the same as in S9 Fig. (TIF) [file pone.0158704.s011.tif]

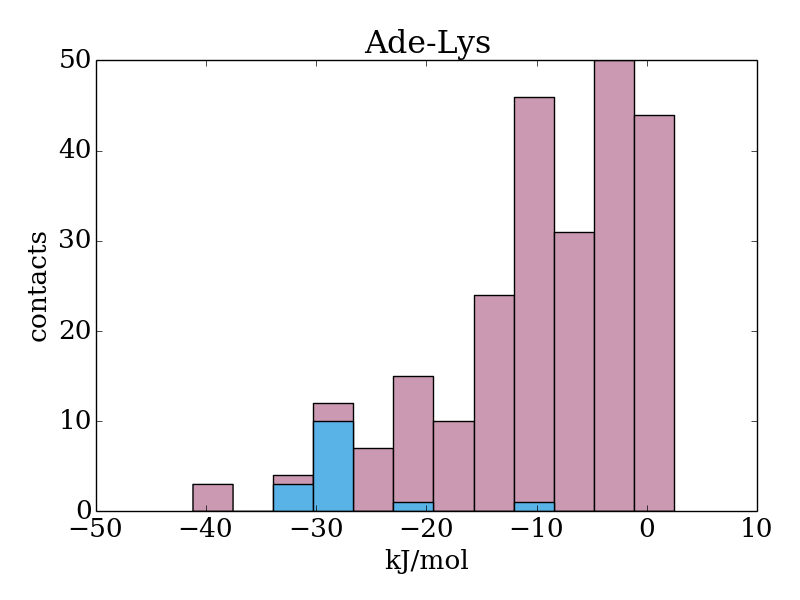

Supplement: S12 Fig — The interaction energies were calculated in an environment with dielectric constant ε = 80. The selection of the data set for the construction of the profile is the same as in S9 Fig. (TIF) [file pone.0158704.s012.tif]

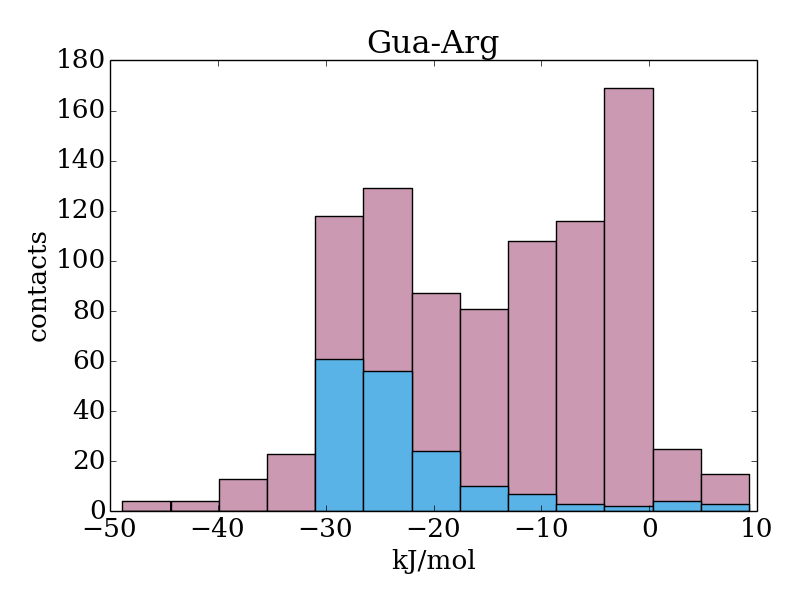

Supplement: S13 Fig — The interaction energies were calculated in an environment with dielectric constant ε = 80. Only those dimers in which the amino acid interacts with the base moiety of the nucleotide were considered in the construction of the interaction energy profile. No two 100% identical proteins were present in the set from which the dimers were extracted. The “envelope” of isoenergetic non-cluster contacts covering the cluster profile is present for the symmetry reasons discussed in the legend of S7 Fig and illustrated in S8 Fig. (TIF) [file pone.0158704.s013.tif]

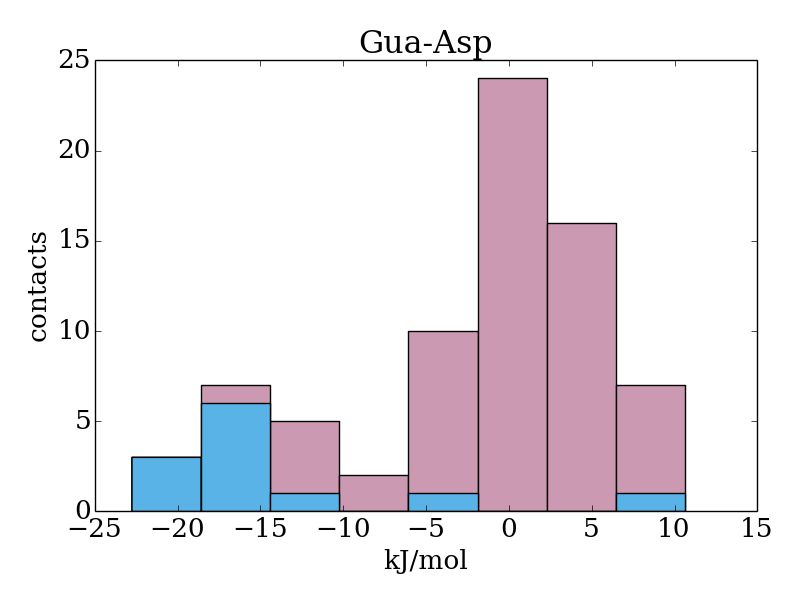

Supplement: S14 Fig — The selection of the data set for the construction of the profile and other computational details are the same as in S13 Fig. (TIF) [file pone.0158704.s014.tif]

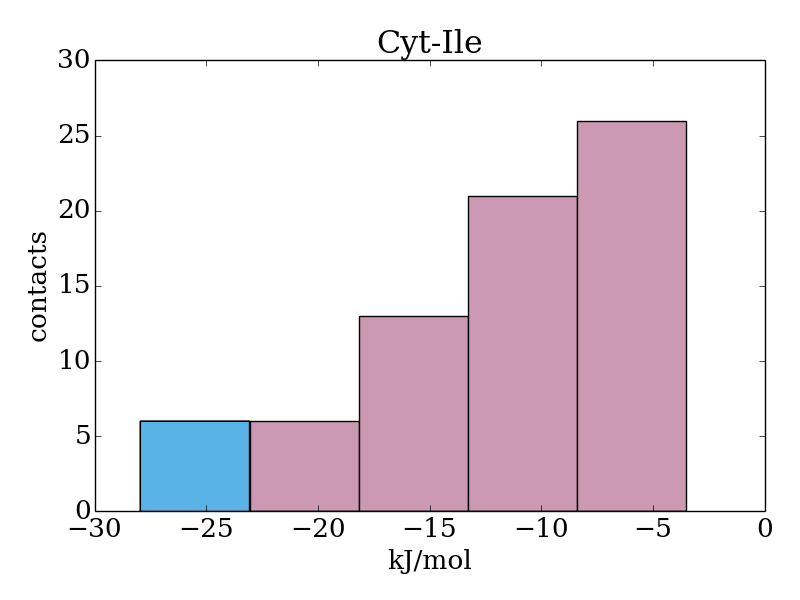

Supplement: S15 Fig — The interaction energies were calculated in an environment with dielectric constant ε = 1. Only those dimers in which the amino acid interacts with the base moiety of the nucleotide were considered in the construction of the interaction energy profile. No two 100% identical proteins were present in the set from which the dimers were extracted. (TIF) [file pone.0158704.s015.tif]

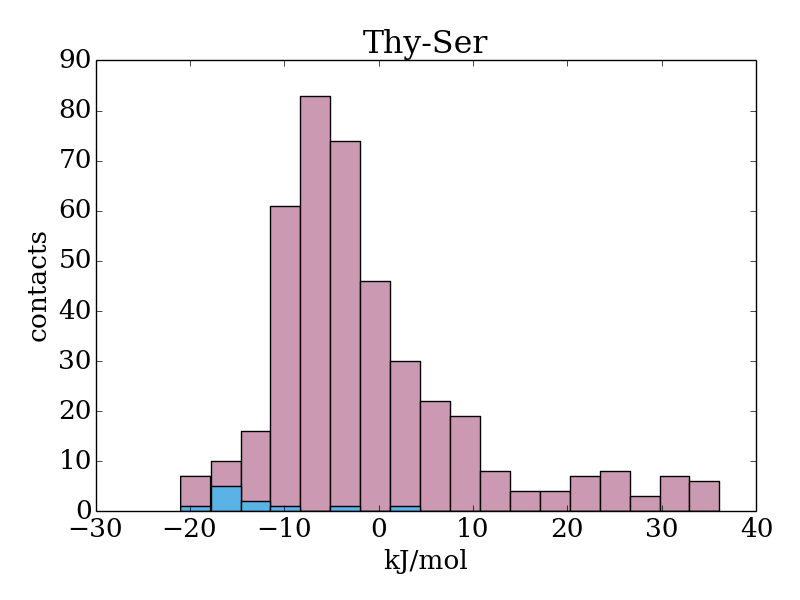

Supplement: S16 Fig — The interaction energies were calculated in an environment with dielectric constant ε = 4. All amino acid—nucleotide dimers were considered in the construction of the interaction energy profile. No two 100% identical proteins were present in the set from which the dimers were extracted. (TIF) [file pone.0158704.s016.tif]

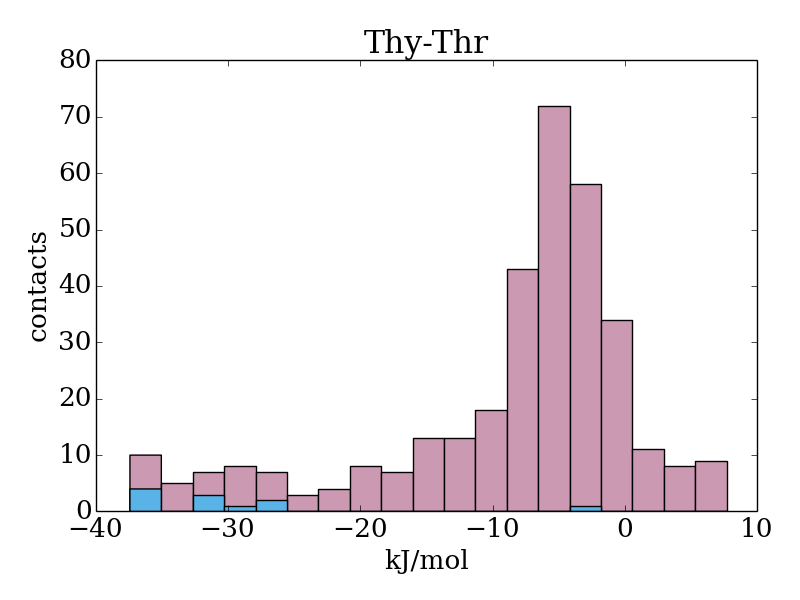

Supplement: S17 Fig — The selection of the data set for the construction of the profile is the same as in S16 Fig. (TIF) [file pone.0158704.s017.tif]

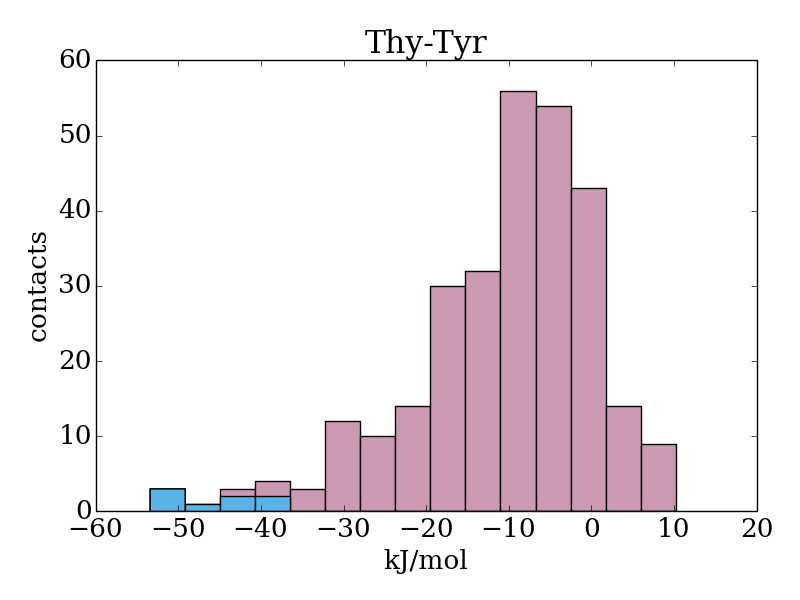

Supplement: S18 Fig — The selection of the data set for the construction of the profile is the same as in S16 Fig. (TIF) [file pone.0158704.s018.tif]

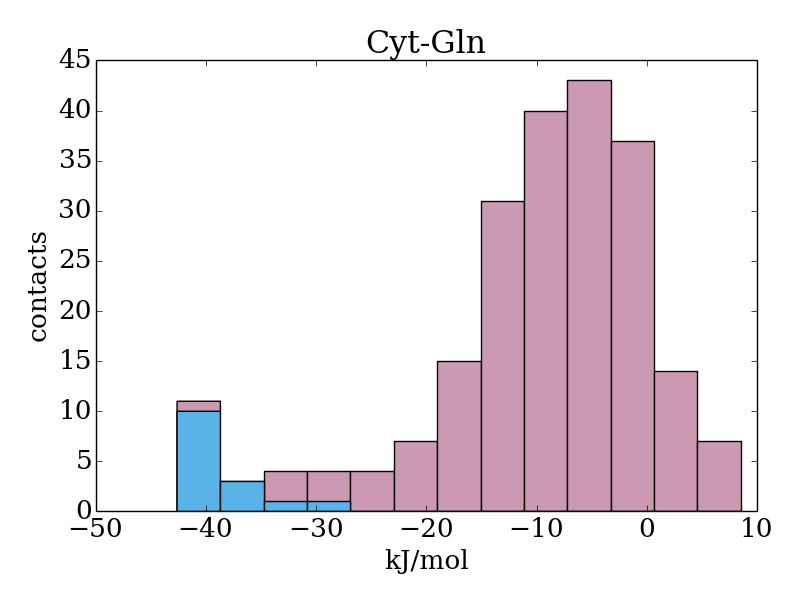

Supplement: S19 Fig — The binding motif realised in the distinctive cluster features only an interaction with the DNA backbone. The interaction energies were calculated in an environment with dielectric constant ε = 4. All amino acid—nucleotide dimers were considered in the construction of the interaction energy profile. No two 100% identical proteins were present in the set from which the dimers were extracted. (TIF) [file pone.0158704.s019.tif]

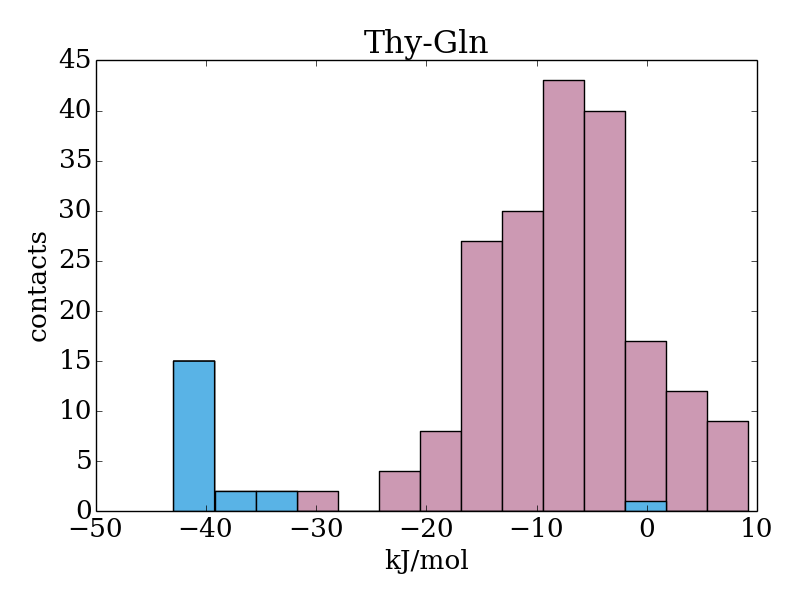

Supplement: S20 Fig — The binding motif realised in the distinctive cluster features only an interaction with the DNA backbone. The selection of the data set for the construction of the profile is the same as in S19 Fig. (TIF) [file pone.0158704.s020.tif]
